# Machine Learning Classification to Identify the Stage of Brain-Computer Interface Therapy for Stroke Rehabilitation Using Functional Connectivity

**DOI:** 10.3389/fnins.2018.00353

**Published:** 2018-05-29

**Authors:** Rosaleena Mohanty, Anita M. Sinha, Alexander B. Remsik, Keith C. Dodd, Brittany M. Young, Tyler Jacobson, Matthew McMillan, Jaclyn Thoma, Hemali Advani, Veena A. Nair, Theresa J. Kang, Kristin Caldera, Dorothy F. Edwards, Justin C. Williams, Vivek Prabhakaran

**Affiliations:** ^1^Department of Radiology, University of Wisconsin-Madison, Madison, WI, United States; ^2^Department of Electrical Engineering, University of Wisconsin-Madison, Madison, WI, United States; ^3^Department of Biomedical Engineering, University of Wisconsin-Madison, Madison, WI, United States; ^4^Department of Kinesiology, University of Wisconsin-Madison, Madison, WI, United States; ^5^Medical Scientist Training Program, University of Wisconsin-Madison, Madison, WI, United States; ^6^Neuroscience Training Program, University of Wisconsin-Madison, Madison, WI, United States; ^7^Deparment of Psychology, University of Wisconsin-Madison, Madison, WI, United States; ^8^Department of Orthopedics and Rehabilitation, University of Wisconsin-Madison, Madison, WI, United States; ^9^Department of Medical Physics, University of Wisconsin-Madison, Madison, WI, United States

**Keywords:** BCI therapy, stroke recovery, functional MRI, functional connectivity, motor network, non-motor networks, machine learning, support vector machine

## Abstract

Interventional therapy using brain-computer interface (BCI) technology has shown promise in facilitating motor recovery in stroke survivors; however, the impact of this form of intervention on functional networks outside of the motor network specifically is not well-understood. Here, we investigated resting-state functional connectivity (rs-FC) in stroke participants undergoing BCI therapy across stages, namely pre- and post-intervention, to identify discriminative functional changes using a machine learning classifier with the goal of categorizing participants into one of the two therapy stages. Twenty chronic stroke participants with persistent upper-extremity motor impairment received neuromodulatory training using a closed-loop neurofeedback BCI device, and rs-functional MRI (rs-fMRI) scans were collected at four time points: pre-, mid-, post-, and 1 month post-therapy. To evaluate the peak effects of this intervention, rs-FC was analyzed from two specific stages, namely pre- and post-therapy. In total, 236 seeds spanning both motor and non-motor regions of the brain were computed at each stage. A univariate feature selection was applied to reduce the number of features followed by a principal component-based data transformation used by a linear binary support vector machine (SVM) classifier to classify each participant into a therapy stage. The SVM classifier achieved a cross-validation accuracy of 92.5% using a leave-one-out method. Outside of the motor network, seeds from the fronto-parietal task control, default mode, subcortical, and visual networks emerged as important contributors to the classification. Furthermore, a higher number of functional changes were observed to be strengthening from the pre- to post-therapy stage than the ones weakening, both of which involved motor and non-motor regions of the brain. These findings may provide new evidence to support the potential clinical utility of BCI therapy as a form of stroke rehabilitation that not only benefits motor recovery but also facilitates recovery in other brain networks. Moreover, delineation of stronger and weaker changes may inform more optimal designs of BCI interventional therapy so as to facilitate strengthened and suppress weakened changes in the recovery process.

## Introduction

Recent advancements in neurotechnology have led to the emergence of the brain-computer interface (BCI), which records neural signals and translates them into signals that can control assistive devices, such as computers or prostheses. To date, BCI-based approaches are being investigated as therapeutic strategies to facilitate recovery for several neurological diseases, including stroke, epilepsy, and Parkinson's Disease. For stroke, the long-term objective of the rehabilitation is to improve impaired brain functions so as to restore autonomy in daily activities for stroke survivors. While conventional approaches such as physical therapy and occupational therapy have proven to be successful in aiding stroke recovery in the acute and sub-acute stages (Bütefisch et al., 1995; Gordon et al., 2004) modern technologies involving robotics (Kwakkel et al., 2008), transcranial magnetic stimulation (Corti et al., 2012), and virtual reality (Lohse et al., 2014) have demonstrated promise in promoting additional motor and cognitive recovery to improve autonomy and overall quality of life for stroke survivors even in the chronic stages. The use of an electroencephalogram (EEG)-based brain-computer-interface (BCI) is an unconventional rehabilitation strategy that has emerged as a potentially effective therapeutic modality for promoting motor recovery in patients with stroke (Silvoni et al., 2011). An EEG-based BCI detects and uses a patient's neural signals as inputs to provide real-time feedback, effectively enabling users to modulate their brain activity (Felton et al., 2009). Additional feedback presented by means of functional electrical stimulation (FES; De Kroon et al., 2002) and tongue stimulation (TS) (Wilson et al., 2012) also provide users with multi-modal feedback as a form of reward for producing certain brain activity patterns while performing tasks. While BCI therapy is often explicitly targeted at restoring motor functions, simultaneous changes in non-motor-related functions in the brain may also result after intervention; to date, neural reorganization of cortical regions outside of the motor network is not well-characterized. Distinction between the overall brain state before and after the therapy could facilitate a more thorough understanding of the mechanisms underlying both the strengthening and/or weakening in motor and non-motor networks in participants. Access to this information could allow us to optimize the design and execution of this therapy for stroke rehabilitation.

While EEG allows for study of real-time brain activity during the BCI therapy with a high temporal resolution, neuroimaging methods have afforded us the ability to study both large-scale and small-scale reorganization of brain networks (Van Den Heuvel and Pol, 2010) at a relatively higher spatial resolution. Resting state functional magnetic resonance imaging (rs-fMRI), specifically, has been demonstrated as a powerful and attractive tool to study changes in brain functions as it is non-invasive, time-efficient, and task-free. Rs-fMRI allows us to measure the temporal correlation of the spontaneous, low-frequency (<0.1 Hz) blood-oxygen-level dependent (BOLD) signals across regions in the resting brain. Oscillations in the BOLD fMRI signals are indicative of cortical dynamic self-organization and have been associated with the neural reorganization underlying cognitive and motor function during stroke recovery (Lee et al., 2013; Bajaj et al., 2015). Previous studies have demonstrated that there are overlapping networks between the rs-fMRI-derived motor network and those observed during motor imagery and motor execution fMRI tasks (Grefkes et al., 2008; Nair et al., 2015). A growing number of studies have utilized neuroimaging methods to study the efficacy of BCI therapy in stroke recovery and found modulating changes in neuroplasticity and improvement in motor functions (Di Bono and Zorzi, 2008; Várkuti et al., 2013; Song et al., 2014; Young et al., 2014b; Nair et al., 2015; Soekadar et al., 2015). In the present study, we aim to use rs-fMRI to examine changes in neuroplasticity in whole-brain networks and to examine interactions between motor and non-motor cortical regions in chronic stroke participants following BCI therapy.

A whole-brain analysis resulting in high-dimensional data calls for the application of machine learning-based approaches which have become increasingly more integrated in neuroimaging analysis as they enable discovery of multivariate relationships beyond those identifiable by traditional univariate analysis. Several studies have underscored the utility of machine learning to not only differentiate among population groups (Dai et al., 2012; Meier et al., 2012; Rehme et al., 2014; Fergus et al., 2016; Khazaee et al., 2016; Ding et al., 2017) but also make predictions about behavioral outcomes using regression models (Dosenbach et al., 2010; Vergun et al., 2013; Mohanty et al., 2017), all of which have advanced our understanding of altered brain functionalities associated with several neurological diseases. In the context of BCI systems, linear and non-linear machine learning classification algorithms (Muller et al., 2003; Lotte et al., 2007) including support vector machines (SVMs; Rakotomamonjy and Guigue, 2008), nearest neighbors (Mason and Birch, 2000), and neural networks (Cecotti and Graser, 2011) have mainly been limited to improvement and optimization of the BCI2000 system from a design perspective to make the system more adaptive and user-friendly (Selim et al., 2008; Danziger et al., 2009; Alomari et al., 2013). Relatively fewer studies have applied machine learning techniques to elucidate the therapeutic impact of BCI interventional therapy in stroke patients based on the dynamics of brain connectivity changes. Specifically, SVM-based classifiers have demonstrated the ability to not only draw a distinction between different classes but also provide insight into underlying features that lead to the separation between them (Dosenbach et al., 2010; Vergun et al., 2013). Given that we aim to extensively investigate whole-brain effects of BCI therapy, a similar classification approach is befitting due to its efficiency in handling high-dimensional rs-fMRI data. Recent developments have brought deep learning approaches into view with applications in the field of medical imaging such as tissue/lesion/tumor segmentation (Birenbaum and Greenspan, 2016; Kamnitsas et al., 2017), image reconstruction/enhancement (Benou et al., 2016; Hoffmann et al., 2016) and population-based classification (Brosch et al., 2013; Payan and Montana, 2015). The efficiency of deep learning algorithms, however, is highly dependent on samples available for training a reliable model. Thus, we adhere to supervised machine learning classifiers given the limited sample size.

With the above considerations in mind, the goal of this study was to identify the stage of therapy using whole brain rs-fMRI data in stroke participants undergoing EEG-based BCI intervention along with additional feedback provided by FES and TS. We analyzed changes in non-motor regions of the brain in addition to the well-studied motor regions following BCI therapy in chronic stroke participants. To this end, we modeled this as a classification problem of discriminating between pre-therapy and post-therapy stages of intervention. Specifically, we illustrated using rs-fMRI that connectivity at the pre-therapy stage can be differentiated from that at post-therapy with reasonable accuracy. A SVM-based machine learning classifier was employed to identify specific functional nodes and connections in the brain between the two stages. The significance of this study is 4-fold: this study suggests that (i) a 10-min task-free rs-fMRI scan could aid in identifying and tracking changes in functional connectivity in the brain over the course of BCI therapy; (ii) SVM-based classification can automate the process of categorizing participants into pre-therapy or post-therapy stages and identify features discriminating between the stages of therapy; (iii) BCI therapy, targeted toward upper-extremity motor restoration, can promote recovery effects related to brain connectivity in both motor and non-motor networks; (iv) identification of specific functional changes that strengthen and weaken between stages of BCI-therapy could inform more tailored designs of BCI systems that facilitate stronger changes and suppress weaker changes to maximize the efficacy of this interventional therapy and improve outcomes for stroke survivors.

## Methods

### Study design

**Figure 1:**
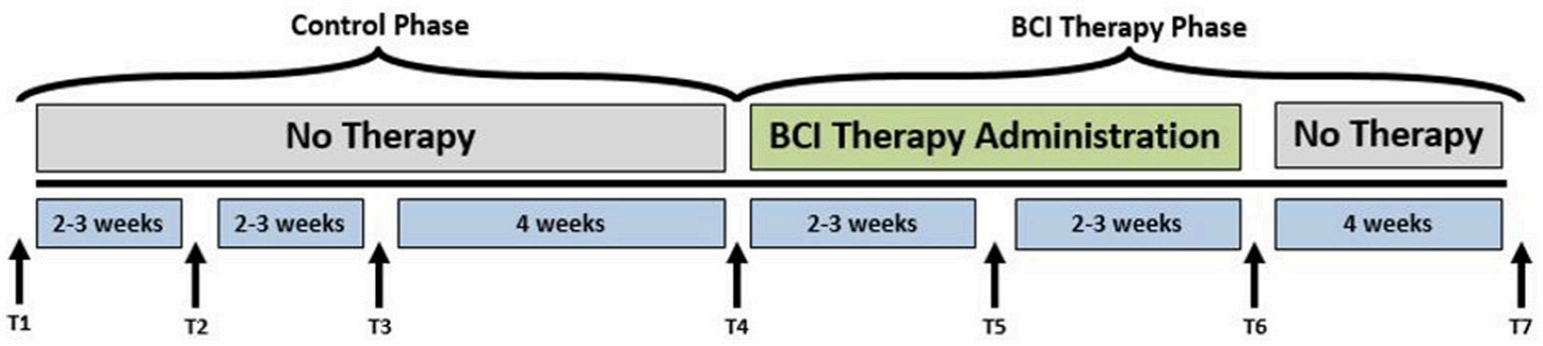
Study paradigm. The time-points at which neuroimaging data were collected are represented by: T1: Control baseline 1, T2: Control baseline 2, T3: Control baseline 3, T4: Therapy baseline T5: Mid-therapy, T6: Post-therapy, and T7: 1-month post-therapy. While the crossover control group completed visits T1 through T7, the BCI therapy group completed visits T4 through T7 only.

A permuted-block design (Zelen, 1974) that accounted for participant characteristics such as gender, stroke chronicity, and severity of motor impairment was used to randomly assign participants to one of two groups: crossover control group and BCI therapy group. The study paradigm is schematized in Figure 1. Ten participants in the BCI therapy group received interventional rehabilitation therapy and were scanned for MRI and rs-fMRI at four time points: pre-therapy (T4), mid-therapy (T5), immediately post-therapy (T6), and 1 month after completing the last BCI therapy (T7) as per the figure. Ten participants in the crossover control group first received three functional assessments and MRI scans during the control phase in which no BCI therapy was administered (T1 through T3 in Figure 1), and their assessments were spaced at intervals similar to those given during the BCI therapy phase. Upon completion of the control phase of the study, the crossover control group “crossed over” into the BCI therapy phase of the study. For this study, participants from the crossover control group and the BCI therapy were combined (*N* = 20), treated as a single sample group and studied at the pre-therapy (T4) and post-therapy (T6) stages to provide additional power to the analysis. Even though imaging data were collected at four distinct time-points, changes between pre-therapy and post-therapy were examined as maximal changes would be expected to occur between these two time-points. Therefore, results from this study should be used to demonstrate proof-of-concept.

### Participants

All participants were recruited as part of an ongoing stroke rehabilitation study to investigate the effects of interventional therapy using an EEG-based BCI device targeting upper extremity motor function. The inclusion criteria for participation were: (1) at least 18 years of age; (2) persistent upper extremity motor impairment resulting from an ischemic or hemorrhagic stroke; (3) ability to provide written informed consent. Exclusion criteria consisted of: (1) concomitant neurodegenerative or other neurological disorders; (2) psychiatric disorders or cognitive deficits that would preclude a participant's ability to provide informed consent; (3) pregnant or likely to become pregnant during the study; (4) allergies to electrode gel, metal and/or surgical tape, contraindications to MRI; (5) concurrent treatment for infectious disease. The study was approved by the University of Wisconsin-Madison Health Sciences Institutional Review Board. All participants provided written informed consent for participation prior to the start of their participation in the study. Participant age was reported corresponding to the first session of BCI therapy. This analysis was limited to chronic stroke participants only (time between stroke onset and the first session of BCI therapy >6 months) since participants in the acute or sub-acute stages often exhibit spontaneous post-stroke recovery that may prove difficult to distinguish from the effects of BCI therapy. While stroke severity was evaluated based on NIH Stroke Scale (NIHSS) score (Brott et al., 1989), the severity of motor impairment was assessed on the basis of standardized scores on the Action Research Arm Test (Carroll, 1965; Lang et al., 2006) and was dichotomized into severe and moderate. Group participant characteristics are summarized in Table 1.

**Table 1:** Study sample characteristics.

**Characteristic**	**Value**
Sample size	20
Age (mean age ± SD)	62.4 ± 14.3 years
Gender (male/female)	12/8
Lesion hemisphere (left/right)	8/12
Time since stroke (mean ± SD)	37.6 ± 40.8 months
Stroke severity (severe/moderate)	11/9

### BCI therapy

The primary purpose of using BCI therapy in this work was to promote restorative function by providing neuromodulatory training with concurrent assistive stimulation that generated actual movement in the impaired upper limb. The BCI device was controlled by actual attempted movement of the user and not imagined movement. The attempted movement, in turn, generated neural activity, as recorded by EEG signals, which translated into computer-generated feedback in real time. Here we provide a concise summary of the procedure for the BCI intervention. The steps of intervention were consistent with those described in depth in prior studies (Wilson et al., 2009; Young et al., 2014a). Neural activity was recorded using a 16-channel EEG cap (g.GAMMA cap, Cortech Solutions) and amplifier (Guger Technologies) and processed using BCI2000 software (Schalk et al., 2004). Movements of the impaired upper extremity were facilitated with two forms of external stimulation: TS (TDU 01.30, Wicab Inc.) and FES (LG-7500, LGMedSupply; Arduino 1.0.4). Three main components were implemented: (i) open-loop attempted movement without any feedback for determination of channels and frequencies for subsequent steps; (ii) closed-loop attempted movement with visual feedback in the form of a cursor task that utilized EEG signals of the user in real time; and (iii) closed-loop attempted movement as in step (ii) with additional feedback in the form of TS and FES to the muscles of the impaired arm.

### Data acquisition: neuroimaging data

Structural MRI scans lasting about 5 min were acquired on 3T GE 750 scanners (GE Healthcare, Waukesha, WI) equipped with an eight-channel head coil. These were T1-weighted axial anatomical scans and were collected using FSPGR BRAVO sequence with the following specifications: *TR* = 8.132 ms, *TE* = 3.18 ms, *TI* = 450 ms over a 256 × 256 matrix and 156 slices, flip angle = 12°, FOV = 25.6 cm, slice thickness = 1 mm. Ten-minute rs-fMRI were collected with participants lying in the scanner with their eyes closed. Participants were instructed to relax with their eyes closed while trying not to fall asleep during this scan. Rs-fMRI scans were obtained using single-shot echo-planar T2^*^-weighted imaging with the following parameters: *TR* = 2.6 s, 231 time-points, *TE* = 22 ms, FOV = 22.4 cm, flip angle = 60°, voxel dimensions 3.5 × 3.5 × 3.5 mm^3^ and 40 slices.

#### Data availability statement

The raw data supporting the conclusions of this manuscript will be made available by the authors, without undue reservation, to any qualified researcher.

### Individual participant analysis

#### Data preprocessing

All scans were inspected visually to ensure they were free of any apparent artifacts. Rs-fMRI data were processed using Analysis of Functional NeuroImaging (AFNI) (Cox, 1996) software. Functional scans were despiked, slice time corrected, motion corrected, aligned with the anatomical scan, normalized to the standard MNI (Montreal Neurological Institute) space using the T1 scan, resampled to 3.5 mm^3^, and spatially smoothed with a 4-mm full-width-half-maximum Gaussian kernel. Motion censoring (per TR motion >1 mm or 1°), regression of white matter and cerebrospinal fluid signals, and bandpass frequency filtering were performed simultaneously in one regression model. The bandpass filtering was focused to the typical low oscillation fluctuations within 0.01–0.1 Hz. Global signal regression was omitted due to ongoing controversy in the literature associated with its use (Murphy and Fox, 2016).

#### Seed-based functional connectivity

**Figure 2:**
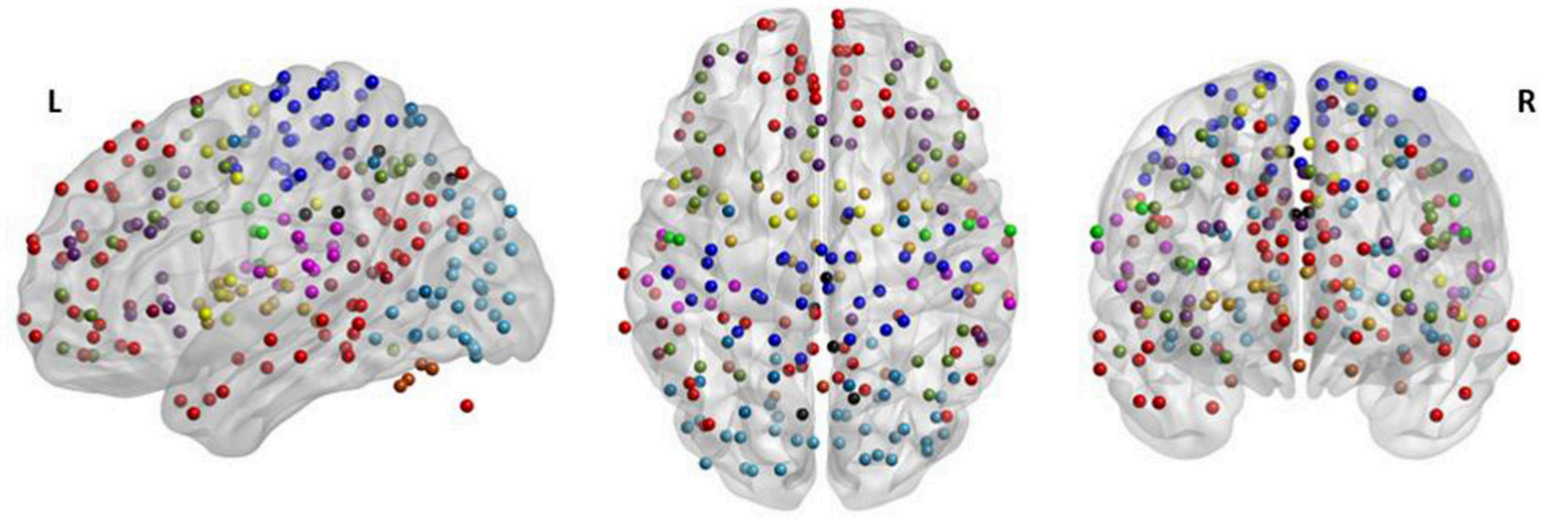
The 236 seeds regions involving motor and non-motor regions include 13 major brain networks color coded according to Table 2 and visualized using BrainNet Viewer (Xia et al., 2013). The seed regions falling outside the template of cerebrum were part of the cerebellum.

**Figure 3:**
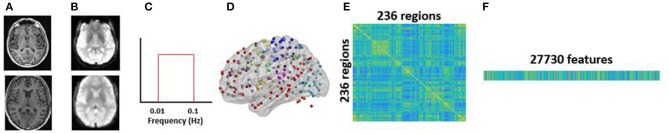
Methodology for single-participant analysis: **(A)** raw structural T1 scan (top) was preprocessed and spatially normalized to MNI space (bottom); **(B)** raw functional scan (top) was preprocessed up to smoothing (bottom); **(C)** smoothed fMRI was temporally filtered to obtain the low frequency oscillations within the range of 0.01–0.1 Hz using a bandpass filter; **(D)** 236 seeds comprising of 13 major brain networks were used to extract BOLD time courses at each seed region; **(E)** 236 × 236 rs-FC matrix was computed using the BOLD time courses; **(F)** unique pairwise correlations contained in the lower triangle of the rs-FC matrix were extracted and vectorized into a 27,730-dimensional vector.

Based on a previous study (Power et al., 2011), 236 seed regions of interest (ROI) spanning regions from 13 distinct networks were selected. This seed template provides full coverage of various motor and non-motor brain regions and has been utilized to study functional reorganization of the brain in healthy participants. The regions are depicted in Figure 2, as per the MNI coordinates, and the networks are encoded as per Table 2. Spherical seeds of 5 mm radius each were created for each participant. This seed template was applied to the spatially normalized, smoothed, and filtered residuals of the resting data and BOLD time series was extracted at each of the 236 seed regions. A correlation matrix of size 236-by-236 was generated by temporally correlating time series from all pairs of seeds. Of the 55,696 correlation coefficients generated, 27,730 unique coefficients were retained for analysis and the duplicates were discarded. The unique correlation coefficients were computed from data at the pre- and post-therapy stages and used as input features for the discrimination between the stages. The methodology at single-participant level is outlined in Figure 3.

**Table 2:** The seed template encompasses the whole brain comprising of 13 distinct brain networks coded by colors and specified number of regions.

**Brain network**	**Seed color**	**Number of seeds**
Sensory/somatomotor hand		30
Sensory/somatomotor mouth		5
Cingulo-opercular task control		14
Auditory		13
Default mode		58
Memory retrieval		5
Ventral attention		9
Visual		31
Fronto-parietal task control		25
Salience		18
Subcortical		13
Cerebellar		4
Dorsal attention		11

### Group level analysis

Applications of classification using machine learning algorithms such as SVM on rs-fMRI have been demonstrated in multiple studies (Dosenbach et al., 2010; Vergun et al., 2013). For the purpose of this study, we adopted a similar strategy, i.e., we applied a binary linear-kernel SVM to rs-FC in order to classify between the two classes, namely pre-therapy and post-therapy. The rs-FC data for all participants were aggregated and the steps described as follows were implemented.

#### Outlier removal

It is acknowledged that with a limited sample size, the data could be skewed due to the presence of outliers; therefore, possible outlier features were detected and removed from the data set. To this end, a median absolute deviation (MAD) (Leys et al., 2013) method detected any value that is more than three scaled MADs away from the median in a given feature which is deemed an outlier. This was repeated for each feature within the pre-therapy stage and post-therapy stage. The features containing these outliers were eliminated, saving only common features across pre- and post-therapy.

#### Feature selection and transformation

The rs-FC per participant consisted of 27,730 coefficients resulting in a high-dimensional dataset. Drawing useful conclusions based on a reasonable classifier is incumbent upon selecting meaningful and important features. One way to achieve this is by means of dimension reduction. Given that a large number of features with a small sample size can result in overfitting to noise, we adopted a feature selection step followed by a feature transformation step. The feature selection was a preprocessing step to select a subset of 27,730 features using a univariate paired *t*-test between the features of pre-therapy and post-therapy stages. Features were tested for normality using the Kolmogorov-Smirnov test (Massey, 1951) and a subset of normal features was selected on the basis of the *p*-value for each individual feature that indicated its effectiveness in the separation between the two aforementioned stages. However, the filtered features were still high-dimensional and could easily lead to overfitting. Therefore, the reduced data obtained from the previous step were transformed to a lower dimensional space using principal component analysis (PCA; Jolliffe, 1986; Jackson, 2005). A PCA-based feature transformation was suitably chosen as it assumes that data can consist of correlated variables (features) and the redundancy can be simplified by forming an uncorrelated basis composed of the principal components which is low-dimensional and accounts for a large fraction of variance in the original data. Each principal component is simply a linear combination of the original rs-FC features. PCA is based upon computation of covariance matrix of the raw data. Only mean centering was applied to the raw data prior to application of PCA. Variance was not standardized as it can change the covariance matrix and lead to misleading principal components. The first few principal component scores were selected based on the amount of variance accounted for in the raw data and were used in the classification step.

#### Classification

Once the appropriate number of principal components was extracted in the feature selection and transformation step, classification between the pre-therapy and post-therapy stages was performed using the learned principal component-based features. The inputs to the classifier were no longer the raw rs-FC coefficients. Instead, the principal component scores, each of which corresponded to a linear combination of multiple rs-FC features, were fed into the classifier as features. Additionally, since SVM-based classifiers do not assume data to be normally distributed, the traditional Fisher z-transformation was not necessary. However, the principal component scores were scaled and standardized so that each component score had the same mean and variance to avoid some features from potentially dominating others due to large magnitude. This was realized by mean centering and scaling by the standard deviation of each component score. A binary classifier was trained on these features and cross-validated on an out-of-sample participant. To allow for more straightforward interpretation of results, a linear-kernel SVM was applied due to the advantage of ease of interpretation of results. Additionally, the choice of a linear-kernel classifier was supported by the linear separability in the data. As observed in three-dimensional space in **Figure 5**, the principal component features are almost linearly separable. Thus, there is a likelihood that the two classes are linearly separable in higher dimensions which are used for classification (Noble, 2006).

#### Cross-validation

A leave-one-out cross-validation (LOOCV) method (Hastie et al., 2001) was adopted to estimate classifier performance as it provides an approximation of the test error with lower bias and is more suitable for a dataset with a small sample size such as here. Since our analysis followed a within-participant design, we performed a LOOCV by participant to avoid introducing possible “twinning” bias. This means that the data consisting of 40 observations (pre-FC and post-FC from 20 participants) were subdivided into 20-folds such that each fold comprised of pre-FC and post-FC data from a single participant. The classifier was trained using features from 19-folds (equivalent to 38 observations from pre- and post-stages of 19 participants) and tested on the left-out fold (2 observations from pre- and post-stages of 1 participant). This was repeated 20 times such that data from each participant was left out once while a model was generated using the rest of the data. The performance of the model was assessed by averaging the accuracies over all iterations.

#### Model parameter optimization

To achieve high classification accuracy, the SVM classifier relies on both feature selection and learning optimized model parameters. Specifically, the misclassification cost and kernel scale parameters of the classifier were optimized with a Bayesian optimization (Snoek et al., 2012) approach. By minimizing the cross-validation error over a range of values for 30 iterations, the optimal parameter values were obtained that further improve the classification performance.

#### Feature contribution

Once a model was learned with the optimal parameters, the use of a linear-kernel SVM allowed understanding of underlying discriminatory brain connections. The PCA feature transformation yielded linear coefficients that weigh features and the importance of each feature was dependent upon the magnitude of the associated coefficient.

#### Seed contribution

Based upon the feature weights obtained for each of the discriminating functional connections, seed region weights were calculated for individual brain regions. This was achieved by halving the feature weight of each functional connection and assigning this value to the two seeds involved (Meier et al., 2012). A cumulative measure of weight corresponding to each seed was computed by averaging the half-weights across all discriminating connections.

#### Overview of methodology

**Figure 4:**
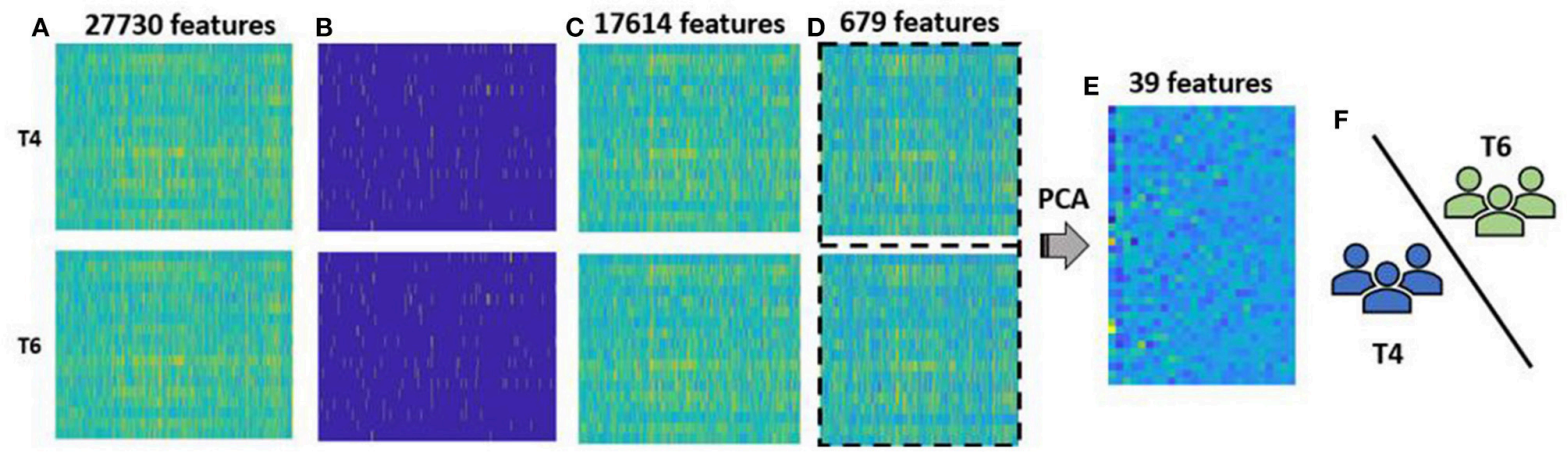
Methodology for group-level analysis: **(A)** vectorized form of rs-FC matrix for each participant aggregated for T4, i.e., pre-therapy and T6, i.e., post-therapy time points. Each group had 20 participants with 27,730-dimesional features; **(B)** outliers (marked in yellow) at pre- and post-therapy were identified using MAD approach; **(C)** reduced rs-FC matrix after cumulative outliers were removed, i.e., each stage consisted of 20 participants and 17,614 features; **(D)** 679 features that were significantly different between pre- and post-therapy stages as identified by a paired *t*-test were retained and data across the two stages were combined together for a feature transformation step; **(E)** feature transformation using PCA was performed that resulted in data with 40 participants and 39 low-dimensional principal components features. Of them 25 features accounted for more than 85% variance and were used as final features for classification; **(F)** the selected features were fed to the binary SVM classifier that labels each test participant to either pre-therapy or post-therapy stage using LOOCV.

Overall, a classification model using rs-FC was learned and optimized, and the contributing rs-FC features and ROIs that provided the maximum discriminative power based on cross-validation performance were identified. All computations were carried out using the Statistics and Machine Learning Toolbox in MATLAB R2017a (The MathWorks, Inc., Natick, Massachusetts, United States). The group-level analysis pipeline is illustrated in Figure 4.

## Results

### Performance of classifier

#### Outlier removal

Each of the 27,730 features was tested for the presence of outliers within the pre- and post-therapy stages separately. Features were removed if they contained values that were more than three scaled deviations from the median. MAD was chosen as it is more robust in comparison to the standard deviation measure. Outliers constituted 21.99% of the features in the pre-therapy stage and 19.53% of the features in the post-therapy stage. After outliers across both time-points were removed, 17,614 features were retained in each class.

#### Feature selection and transformation

**Figure 5:**
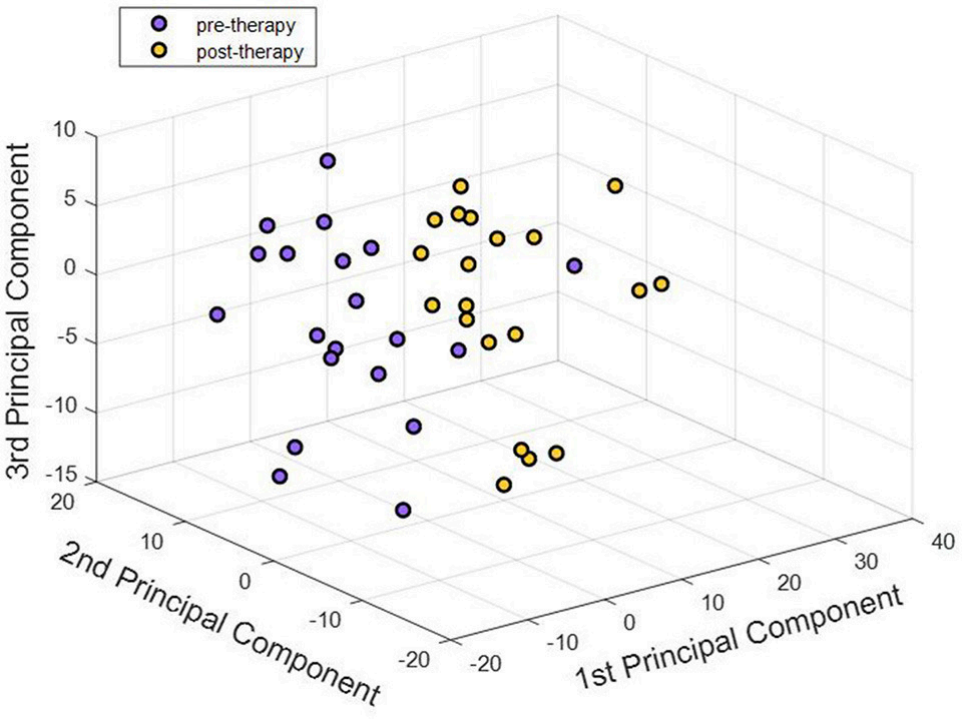
First three principal components corresponding to pre-therapy rs-FC and post-therapy rs-FC for all participants were visualized. Each point in the 3-D plot corresponds to a participant. There appeared to be an almost clear separation between the two stages just with three principal components. Adding higher number of components better explained the variance in the data. Our analysis used 25 components that explained over 85% of the variance in the dataset.

**Figure 6:**
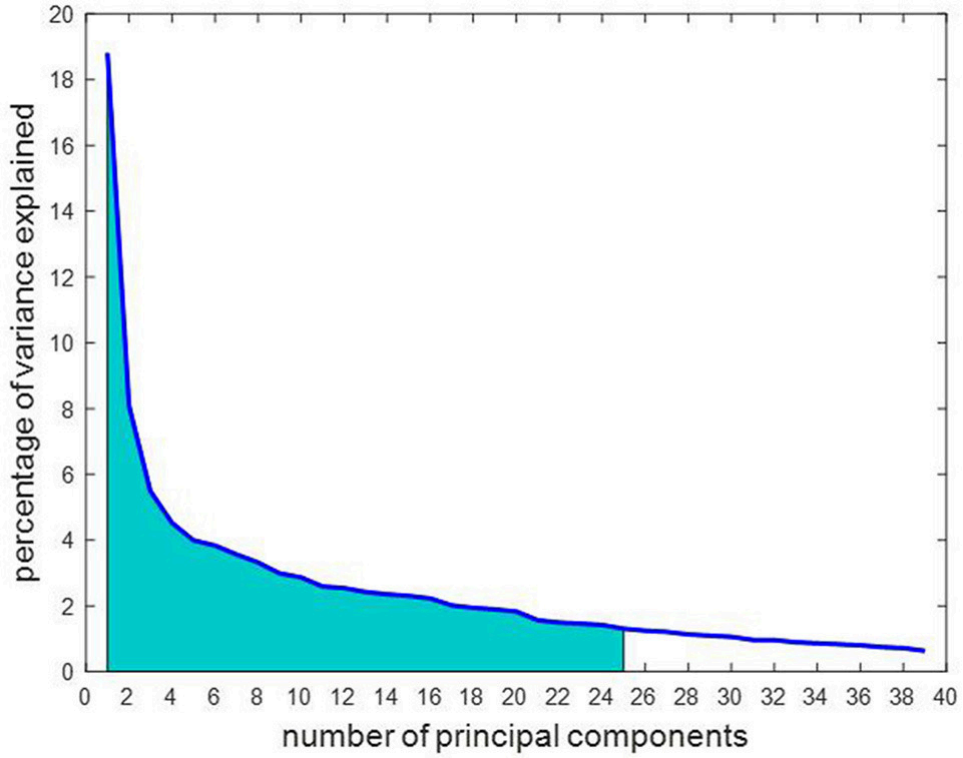
The number of principal components are arranged in order of importance so that the first component accounts for the largest proportion of variance in the rs-FC data. Of the 39 principal components, 25 were chosen as marked in the graph as they cumulatively explained over 85% of the variance in the data, represented by the shaded area under the curve.

The 17,614 features remaining after outlier elimination were used as input to the feature selection step. Each feature was tested for normality and the univariate paired *t*-test resulted in 679 features that were significantly different between the two stages. During feature transformation using PCA, the number of principal components was determined to be the smaller of these two: number of samples-1 or number of input features. Thus, application of PCA resulted in 39 principal components in this case, each of which was uncorrelated to each other and was realized as a linear combination of the 679 input features. Of the 39 components, 25 components were able to account for over 85% of the variance in the data and were fed into the classifier. Due to lack of visualization tools in 25 dimensions, a simpler plot with the first three components was generated as displayed in Figure 5. The separation observed in the visualization suggests that PCA was able to build useful low-dimensional features that can help in differentiating between the two stages. For classification, the chosen number of components was based on the variance explained by them as shown in Figure 6. An account of number of features retained at each step of processing from original space (i.e., features are rs-FC coefficients) to reduced space (i.e., features are principal components) is provided in Table 3.

**Table 3:** The number of features derived from the rs-FC data utilized in various steps of the analysis.

**Analysis step**	**Number of features**	**Feature space**
Original features	27,730	rs-FC
After outlier removal	17,614	rs-FC
After univariate filtering	679	rs-FC
After principal component analysis	39	reduced
Chosen principal components for classification	25	reduced

*The feature space indicates whether the corresponding features were measures of functional connectivity, i.e., rs-FC space or principal components comprised of linear combination of multiple rs-FC features, i.e., reduced space*.

#### Cross-validation

**Figure 7:**
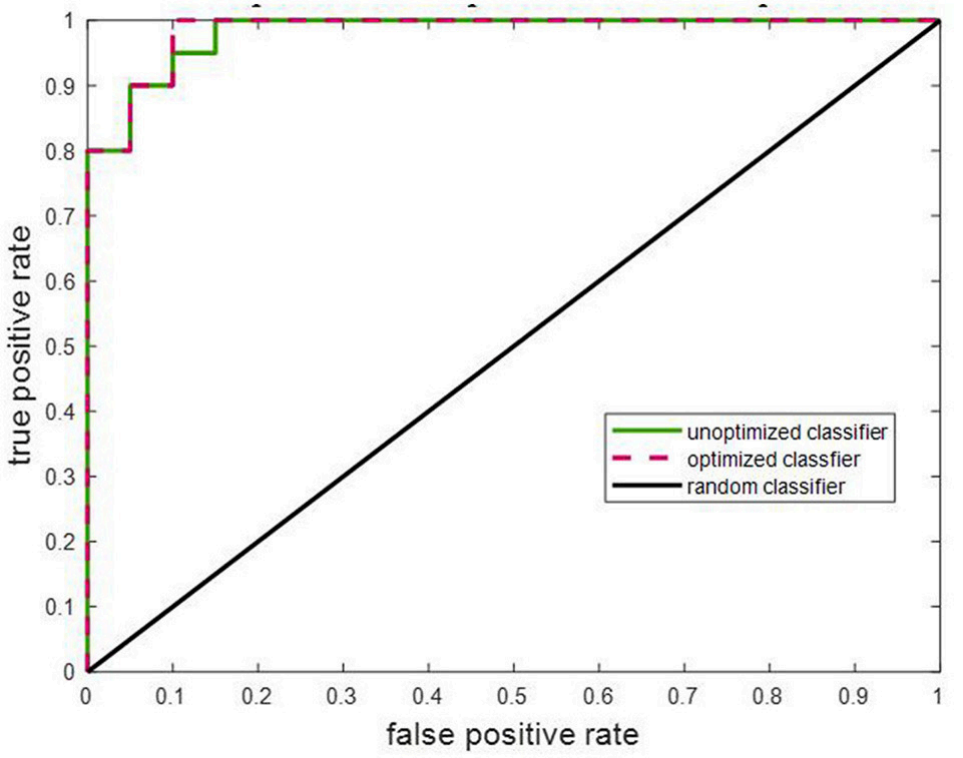
The ROC for the learned SVM classifier was compared to that of a random classifier. The SVM classifier with optimized model parameters showed the best performance. The area under the curves for unoptimized and optimized SVM are specified in Table 4.

A binary SVM classifier was built using 25 principal component features. Classification performance was cross-validated using the LOOCV method and was used to assess and compare results as quantified in Table 4. The accuracy of LOOCV represents the percentage of individual samples that were correctly classified when left out. Since accuracy is a single-point statistic, the results were further broken down into a confusion matrix metric to understand the bias of the classifier toward each class, if any. In addition, multiple performance evaluation metrics were evaluated such as specificity, sensitivity, and area under the curve. The receiver operator curve (ROC) plotted in Figure 7 indicated that the classifiers developed here have superior performance as compared to a random classifier.

**Table 4:** Overall comparative results obtained from LOOCV of binary SVM classifier.

**Metric**	**Performance without optimization**			**Performance with optimization**		
LOOCV accuracy	90%			92.5%		
Confusion matrix		**Pre**	**Post**		**Pre**	**Post**
	Pre	18	2	Pre	18	2
	Post	2	18	Post	1	19
Specificity	0.90			0.95		
Sensitivity	0.90			0.90		
Area under the curve	0.9825			0.9850		
Misclassification cost	1 (default)			0.0010		
Kernel scale	1 (default)			0.0011		

*The rows of confusion matrix represent the actual class while the columns show the predicted class*.

#### Model parameter optimization

The optimal values of classifier parameters, i.e., the misclassification cost and scaling factor for the linear kernel were generated by the Bayesian approach for each classifier and are listed in Table 4. As observed, optimization of the model parameters improves the classifier performance further. This is also reflected in the ROC plot in Figure 7.

### Strengthened and weakened functional changes as discriminating features

**Figure 8:**
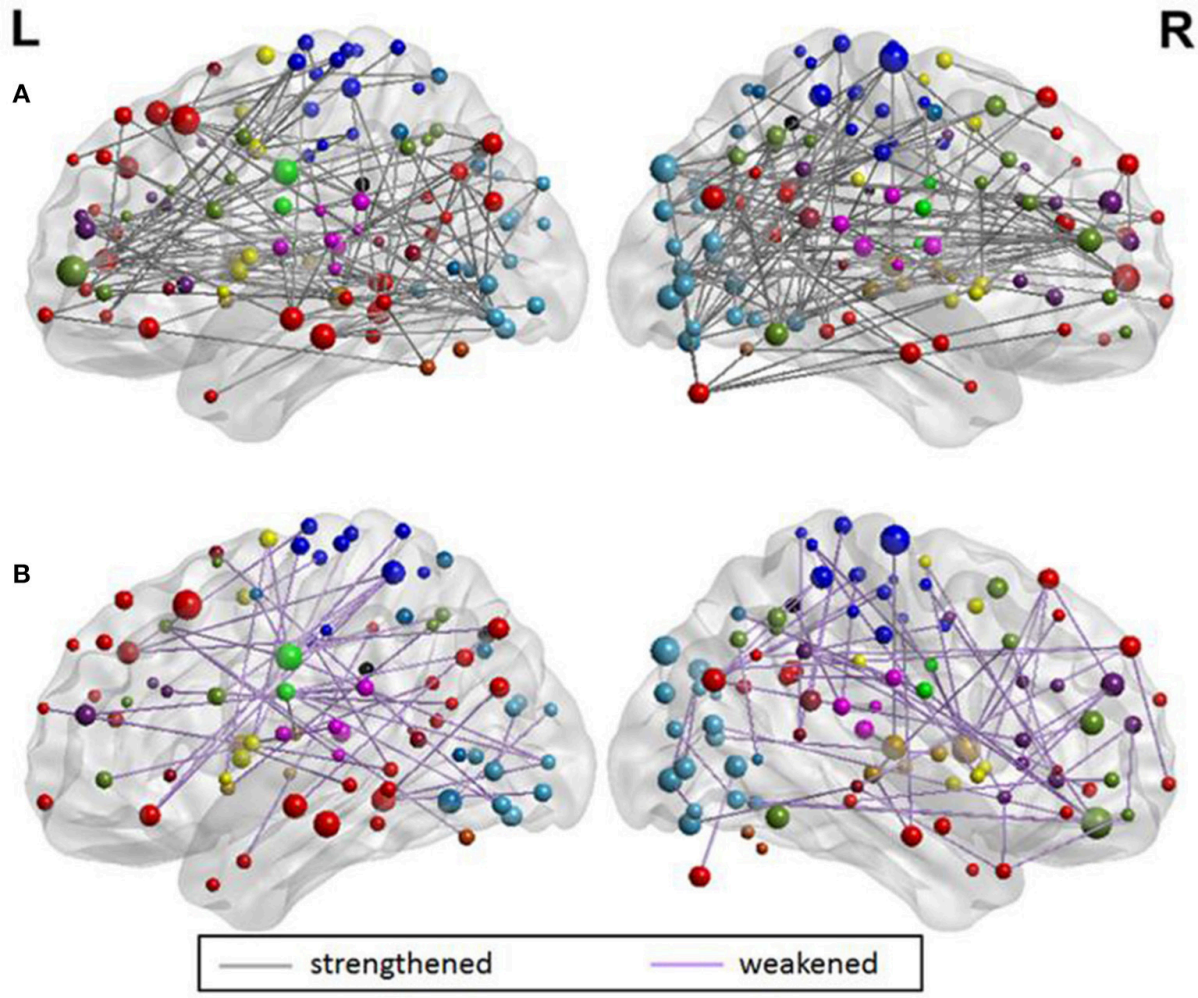
Visualization of **(A)** 441 strengthening functional connections and **(B)** 238 weakening functional connections. The overall number of connections involved in the motor and non-motor networks can be found in Table 5. A detailed list of individual connections can be found in the Supplementary Tables 1, 2, respectively. All brain visualizations were performed using BrainNet Viewer Toolbox (Xia et al., 2013).

From the evaluation of classification performance, it is possible to extract the features that were involved in classification, as well as the importance of each feature in making the distinction between classes. Our objective was to identify discriminating features between groups that strengthened from pre-therapy to post-therapy and those that weakened from pre-therapy to post-therapy. All changes in rs-FC were assessed in terms of group means. Considering the 679 features that went into the final classification model, the distribution of features is presented in Table 5. Stronger connections outnumbered weaker connections in discriminating between the two stages of therapy both in the motor and non-motor networks. Individual functional changes that strengthened and weakened over time are listed in Supplementary Tables 1, 2, respectively in the order of their importance. These changes are also visualized in Figure 8.

**Table 5:** Breakdown of discriminating features into functional connections that strengthened and weakened from pre-therapy to post-therapy are shown for motor as well as non-motor regions.

	**Motor**	**Non-motor**	**Total**
Strengthened	105	336	441
Weakened	71	167	238
Overall	176	503	679

*The colors correspond to the edges in Figure 8. The specific stronger and weaker connections in terms of networks and anatomical locations are provided in Supplementary Tables 1, 2, respectively*.

### Discriminating seed regions

**Figure 9:**
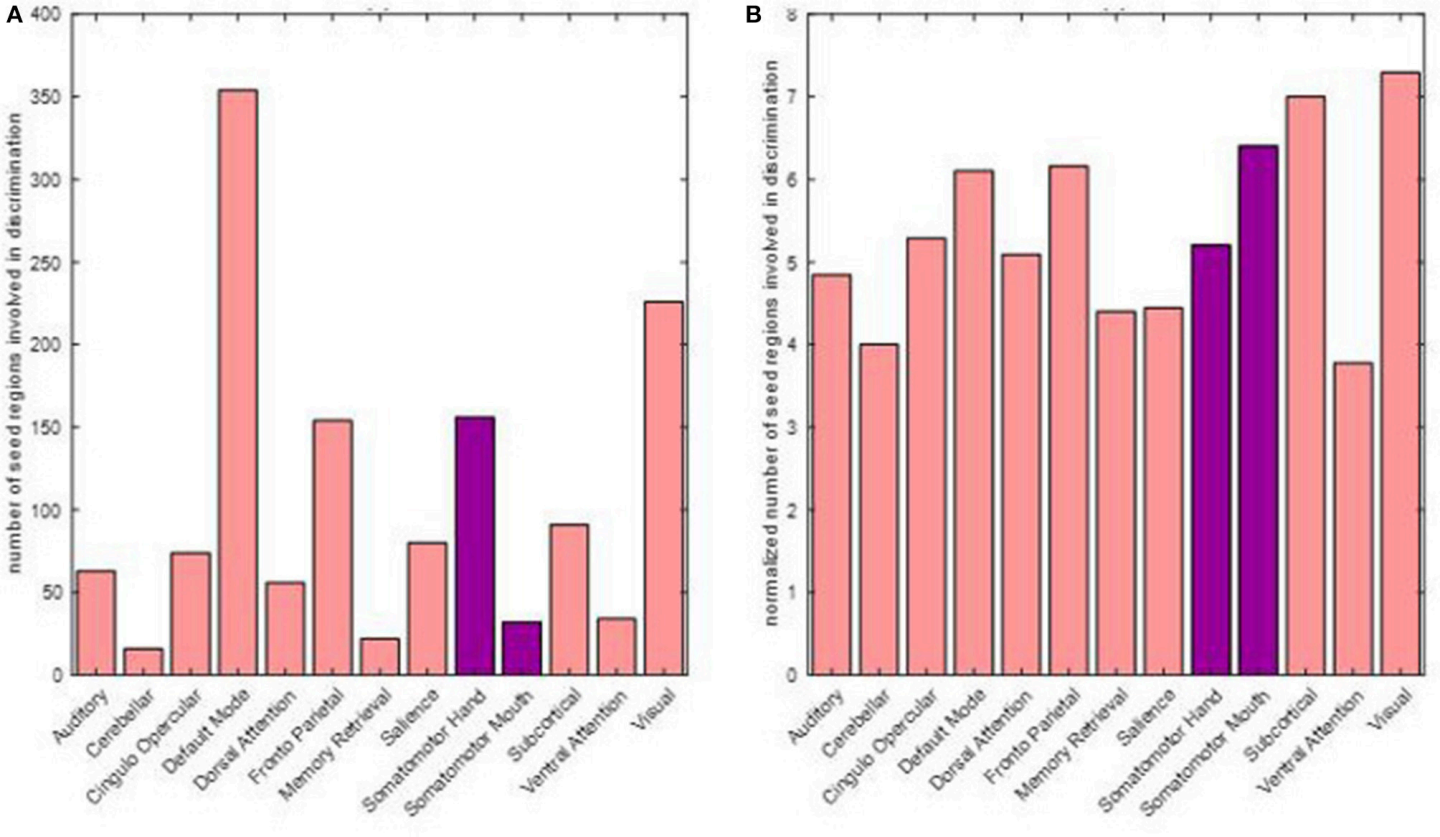
Number of discriminating connections per network is plotted below: **(A)** shows the distribution of involvement of various networks in discriminating features; **(B)** shows the involvement of various networks when normalized with respect to the number of seeds found in each network. The two networks primarily associated with motor functions are highlighted.

Motor as well as non-motor regions were involved in differentiating between pre- and post-therapy. Among the 679 total input features, the distribution of frequency of involved seed regions by network is presented in Figure 9. As observed, seed regions from all major motor and non-motor networks showed involvement in the discriminating features. From Figure 9A, it appeared that the default mode network had the highest number of involved regions; however, the distribution of number of seeds across the networks was not equal as listed in Table 2. The number of discriminating features was normalized by the number of seeds available within each network and plotted in Figure 9B. In particular, networks that exhibited greater normalized involvement included regions from visual, subcortical, fronto-parietal task control, cingulo-opercular task control, default mode, and hand-mouth motor networks.

**Figure 10:**
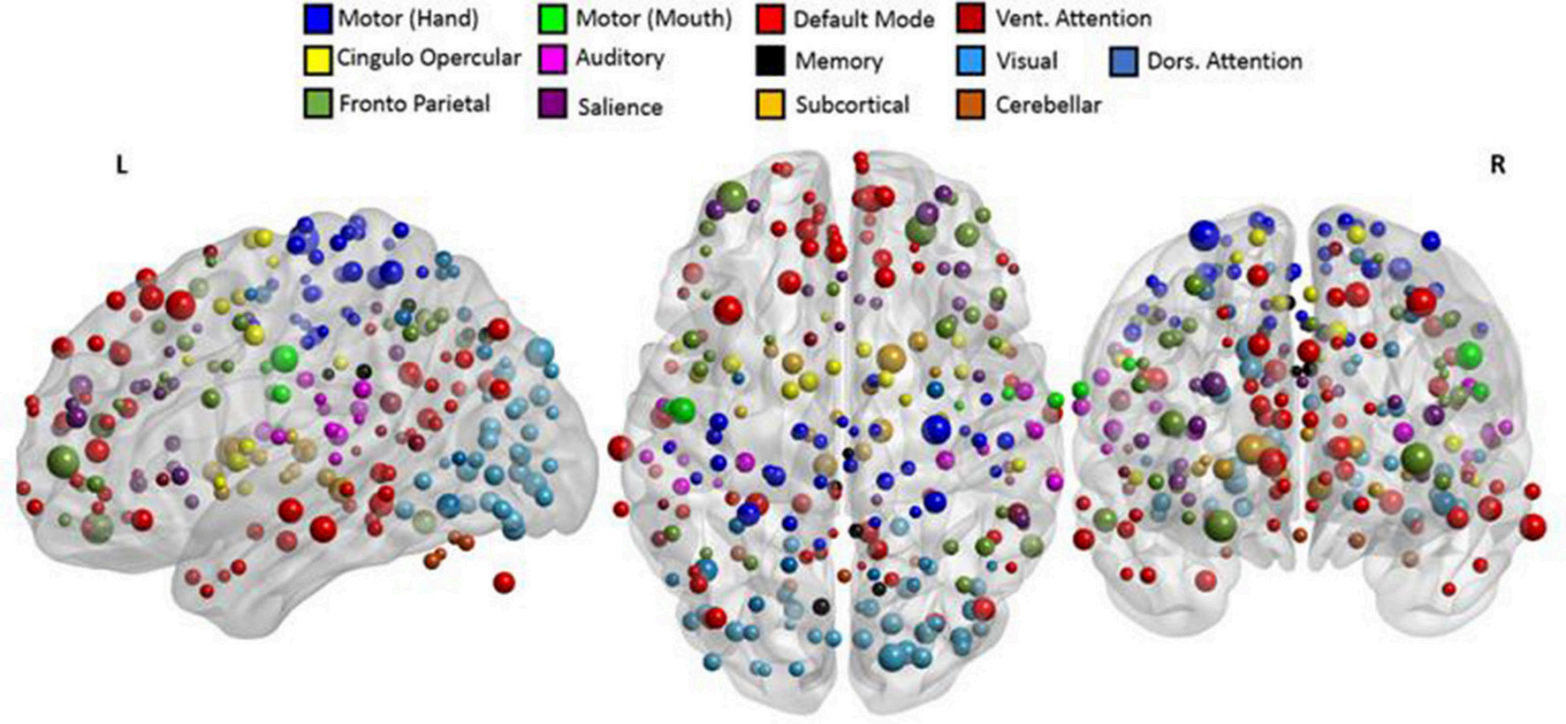
Involved seed regions were weighted as per their contribution in classification. The size of each seed was directly proportional to assigned weight. The top weighted seeds belonged to fronto-parietal, hand motor, default mode, and visual networks. A detailed list of the networks and labels of ROIs ranked as per their weights are presented in Supplementary Table 3.

In addition to assessing the frequency of involvement, the seeds were also assigned weights to study the importance of each seed region based on the coefficients of the principle components. The coefficient corresponding to each feature or connection was halved and assigned to the involved seed regions as per prior work by Vergun et al. (2013). This was repeated across all 25 principal components, and the average of those weights determined the final weight of the seed regions. The weighted seed regions are shown in Figure 10. The complete list of weighted seeds, anatomical locations, and corresponding networks can be found in the Supplementary Table 3. The highly-weighted regions identified are known to be part of the fronto-parietal task control, hand motor, subcortical, visual, and default mode networks.

## Discussion

### Rs-fMRI as a tool to track stroke recovery

Results from this study highlight the utility of rs-fMRI as a tool to track changes in the brain during stroke recovery through rehabilitative therapy. Rs-fMRI is particularly attractive because it only requires about 10 min for acquisition and is task-free. Our analysis suggests that a similar analysis might be extendable to incorporate more than one time-point to gain deeper insight into the recovery process.

### Large-scale impact of BCI stroke rehabilitation

The majority of BCI-aided therapy programs are targeted at the recovery of a particular impairment, such as motor functions, as was the case for participants studied in this cohort. Our findings showed that such a therapy can impact not only motor but also non-motor networks in the brain. We demonstrate a greater number of functional connections growing stronger than ones growing weaker over time over the course of this therapy. These results can better guide the design and implementation of BCI systems to facilitate greater changes that strengthened in patients with stroke.

### Machine learning as a tool to identify stage of therapy and relevant functional differences

As evident from the confusion matrix in Table 4, we were able to differentiate between the two stages of BCI therapy with high cross-validation accuracy. High-dimensional rs-FC extracted from whole brain analysis was downscaled by PCA-based feature transformation that helped elucidate differences across stages of therapy regarding underlying brain connections involved. In comparison to a random classifier that is 50% accurate, our machine learning classifier developed using low-dimensional features derived from rs-FC performed much better with over 90% accuracy. These results indicate that with a large sample size, a SVM classifier could be trained on rs-FC data to categorize a new participant into either the pre-therapy or post-therapy stage of the recovery process by identifying the most discriminative rs-FC features.

### The bigger picture

The current study is presented from a neuroimaging perspective of the changes occurring after BCI therapy. However, other than the neuroimaging methods, EEG and behavioral data are the core components of this interventional study. Since this therapy is based on acquisition of simultaneous EEG, it would be important to understand the spectral data to support the effects of the therapy. Group-level EEG analyses were conducted on the associated cohort (*N* = 21) and the results are currently reported under separate covers to the same issue (Remsik et al., submitted, currently submitted for review to Frontiers in Neuroscience, section Neural Technology). The analysis studied the levels of desynchronization and coherence over the motor cortex and performance with respect to functional outcomes across all time-points. Similarly, rs-FC in the motor cortex before and after the therapy associated with subjective and objective behavioral outcomes have been quantified in another manuscript submitted to the same journal (Mohanty et al., submitted, currently submitted for review to Frontiers in Neuroscience, section Neural Technology).

The most common rehabilitative clinical applications of BCI systems (Bamdad et al., 2015) include speech (Brumberg et al., 2010; Mugler et al., 2013) and motor (Birbaumer, 2006; Neshige et al., 2007; Sun et al., 2011) rehabilitation. Fewer studies have adopted the BCI paradigm for cognitive rehabilitation (Gomez-Pilar et al., 2014). Most of these deal with improving a specific function and study changes occurring in the associated limited brain regions. As per Supplementary Table 3, the motor regions that contributed the most to classification were found over the bilateral precentral gyrus which forms the core of the primary motor cortex. This is in alignment with findings that focus specifically on post-stroke changes in the motor network (Lotze et al., 1999; Young et al., 2014b; Nair et al., 2015). In addition, our study expands the knowledge further by identifying brain changes that occurred in the non-motor areas involving fronto-parietal task control, default mode, and visual networks even though the BCI therapy was primarily targeted at the recovery of motor function. This demonstrates the importance of comprehending the gross impact of BCI therapy on a whole-brain level. Additionally, since the BCI system is adaptive in nature (Schalk et al., 2004), the knowledge about functional changes that are strengthening and/or weakening as a result of this therapy might point toward a better design of the intervention. Maladaptive changes caused by the compensatory activity of the unaffected side has been shown to prevent recovery on the affected side (Takeuchi and Izumi, 2012). One direction to harness this information could involve regulating the way EEG signals are processed within BCI device. The signal processing module of the BCI system that takes into account the signal generated at each output channel could be modulated so as to maximize the changes that grew stronger and minimize the changes that grew weaker, thus, tailoring the therapy for each user.

### Limitations

Our results show that standard machine learning approach has the potential to track recovery through BCI therapy. However, the study was constrained in terms of the sample size since conventional machine learning analysis relies on training on a large dataset so as to have greater power of generalizability. Although we attempted to include a comparable number of participants of both genders, different lesion locations and volumes, and differing levels of stroke severity, heterogeneity in any of these factors might be relevant considerations for future analysis as they could potentially influence the results. In this analysis, the number of samples available for training impacted the number of principal components (rank of covariance matrix) evaluated in the feature transformation step using PCA. Higher number of samples would provide higher degree of freedom. With continuing recruitment, using a larger and more homogeneous participant cohort would allow for more generalizable conclusions. The definition of rs-FC was based upon Pearson's correlation, which is a classical approach and accounts for linear dynamics among the BOLD signals. Recent studies such as that conducted by Smith et al. (2011) provide alternate definitions of rs-FC such as mutual information, cosine similarity, and dynamic time warping; therefore, applying different definitions of seeds and rs-FC could impact the underlying discriminatory features in classification. Although several non-motor networks were identified as being recruited during recovery, we have not investigated the behavioral implications of this finding, i.e., whether strengthened connections in these networks correlate with behavioral gains in various brain functions. The notion of stronger and weaker changes in rs-FC in this study might not reflect adaptive and maladaptive changes in behavioral aspects even though we observed overall improvement at the group-level in measures such as the Action Research Arm Test (mean change = 0.85), and domains of the Stroke Impact Scale (mean change in hand function = 0.75; mean change in physical strength ≤0.13) from pre-therapy to post-therapy.

### Future scope

The ongoing recruitment for this study offers a broad future scope to incorporate more participants that can form a more homogenous cohort. Comparison between stroke participants undergoing rehabilitative therapy and healthy participants undergoing the same therapy will allow comprehension of recovery specifically associated with the event of a stroke. An analysis similar to our study could be extended to incorporate other time-points during the BCI therapy paradigm, such as the mid-therapy (T5) and 1-month post-therapy (T7) time points. Aside from rs-fMRI, alternative neuroimaging methods such as diffusion tensor imaging, task-fMRI, arterial spin labeling, and perfusion imaging capture complementary information and could be used to analyze and compare classification performance.

## Conclusion

We utilized PCA-based feature transformation coupled with a SVM classifier to discriminate stroke participants by stage of BCI intervention (i.e., the pre-therapy stage to the post-therapy stage) on the basis of rs-FC in both motor and non-motor regions. The findings from this study can be summarized as follows: (i) data from a task-free rs-fMRI can help identify changes across stages of the BCI-aided stroke intervention and hence, has the potential to track stroke recovery; (ii) using a machine learning SVM classifier facilitates automation of discrimination between stages of therapy with a reasonably high accuracy and examination of discriminating connections; (iii) both motor and non-motor regions of the brain undergo reorganization during this intervention. Higher number of strengthening functional changes in comparison to the ones weakening between pre- and post-therapy suggests a greater overall positive impact of BCI intervention on stroke recovery at a whole-brain level; (iv) the capability of delineating such specific changes holds promise for better design of the BCI therapy that could incorporate the information by reinforcing stronger changes while suppressing weaker changes.

## Author contributions

RM was involved in data collection, analysis, interpretation of results, and writing of the manuscript. AS was involved in data collection, preprocessing data, and writing the manuscript. BY was involved in subject recruitment, data collection, and editing of the manuscript. AR, KD, TJ, MM, JT, and HA were involved in data collection. VN contributed to data collection, manuscript editing, and intellectual content. TK was involved in the recruitment of study participants. KC was involved in subject recruitment. DE is the co-I and JW, VP are co-PIs, and were involved in study conception, design, manuscript editing, intellectual content, and supervised all aspects of the study.

### Conflict of interest statement

The authors declare that the research was conducted in the absence of any commercial or financial relationships that could be construed as a potential conflict of interest.

## Abbreviations

BCI: brain-computer interface
BOLD: blood-oxygen-level dependent
EEG: Electroencephalography
FES: functional electrical stimulation
LOOCV: leave-one-out cross-validation
MAD: median absolute deviation
MNI: Montreal Neurological Institute
NIHSS: National Institutes of Health Stroke Scale
PCA: principal component analysis
Rs-FC: resting state functional connectivity
rs-fMRI: resting state functional magnetic resonance imaging
SVM: support vector machine
TS: tongue stimulation.

## References

[B1] Alomari M. H. Samaha A. AlKamha K. (2013). Automated classification of L/R hand movement EEG signals using advanced feature extraction and machine learning. *arXiv:13122877*.

[B2] Bajaj S. Butler A. J. Drake D. Dhamala M. (2015). Functional organization and restoration of the brain motor-execution network af ter stroke and rehabilitation. Front. Hum. Neurosci. 9:173. 10.3389/fnhum.2015.00173 25870557 PMC4378298

[B3] Bamdad M. Zarshenas H. Auais M. A. (2015). Application of BCI systems in neurorehabilitation: a scoping review. Disabil. Rehabil. Assist. Technol. 10, 355–364. 10.3109/17483107.2014.961569 25560222

[B4] Benou A. Veksler R. Friedman A. Raviv T. R. (2016). De-noising of contrast-enhanced MRI sequences by an ensemble of expert deep neural networks, in Deep Learning and Data Labeling for Medical Applications, eds Carneiro G. Mateus D. Loïc P. Bradley A. Tavares J. M. R. S. Belagiannis V. Papa J. P. Nascimento J. C. Loog M. Lu Z. Cardoso J. S. Cornebise J. (Athens: Springer), 95–110.

[B5] Birbaumer N. (2006). Breaking the silence: brain–computer interfaces, (BCI) for communication and motor control. Psychophysiology 43, 517–532. 10.1111/j.1469-8986.2006.00456.x 17076808

[B6] Birenbaum A. Greenspan H. (2016). Longitudinal multiple sclerosis lesion segmentation using multi-view convolutional neural networks, in Deep Learning and Data Labeling for Medical Applications, eds Carneiro G. Mateus D. Loïc P. Bradley A. Tavares J. M. R. S. Belagiannis V. Papa J. P. Nascimento J. C. Loog M. Lu Z. Cardoso J. S. Cornebise J. (Athens: Springer), 58–67.

[B7] Brosch T. Tam R. ADN Initiative (2013). Manifold learning of brain MRIs by deep learning, in International Conference on Medical Image Computing and Computer-Assisted, Intervention (Berlin; Heidelberg: Springer), 633–640. 10.1007/978-3-642-40763-5_78 24579194

[B8] Brott T. Adams H. P. Olinger C. P. Marler J. R. Barsan W. G. Biller J.. (1989). Measurements of acute cerebral infarction: a clinical examination scale. Stroke 20, 864–870. 10.1161/01.STR.20.7.864 2749846

[B9] Brumberg J. S. Nieto-Castanon A. Kennedy P. R. Guenther F. H. (2010). Brain–computer interfaces for speech communication. Speech Commun. 52, 367–379. 10.1016/j.specom.2010.01.001 20204164 PMC2829990

[B10] Bütefisch C. Hummelsheim H. Denzler P. Mauritz K.-H. (1995). Repetitive training of isolated movements improves the outcome of motor rehabilitation of the centrally paretic hand. J. Neurol. Sci. 130, 59–68. 10.1016/0022-510X(95)00003-K 7650532

[B11] Carroll D. (1965). A quantitative test of upper extremity function. J. Chronic Dis. 18, 479–491. 10.1016/0021-9681(65)90030-5 14293031

[B12] Cecotti H. Graser A. (2011). Convolutional neural networks for P300 detection with application to brain-computer interfaces. IEEE Trans. Pattern Anal. Mach. Intell. 33, 433–445. 10.1109/TPAMI.2010.125 20567055

[B13] Corti M. Patten C. Triggs W. (2012). Repetitive transcranial magnetic stimulation of motor cortex after stroke: a focused review. Am. J. Phys. Med. Rehabil. 91, 254–270. 10.1097/PHM.0b013e318228bf0c 22042336

[B14] Cox R. W. (1996). AFNI: software for analysis and visualization of functional magnetic resonance neuroimages. Comput. Biomed. Res. 29, 162–173. 10.1006/cbmr.1996.0014 8812068

[B15] Dai Z. Yan C. Wang Z. Wang J. Xia M. Li K.. (2012). Discriminative analysis of early Alzheimer's disease using multi-modal imaging and multi-level characterization with multi-classifier, (M3). Neuroimage 59, 2187–2195. 10.1016/j.neuroimage.2011.10.003 22008370

[B16] Danziger Z. Fishbach A. Mussa-Ivaldi F. A. (2009). Learning algorithms for human–machine interfaces. IEEE Trans. Biomed. Eng. 56, 1502–1511. 10.1109/TBME.2009.2013822 19203886 PMC3286659

[B17] De Kroon J. Van der Lee J. IJzerman M. Lankhorst G. (2002). Therapeutic electrical stimulation to improve motor control and functional abilities of the upper extremity after stroke: a systematic review. Clin. Rehabil. 16, 350–360. 10.1191/0269215502cr504oa 12061468

[B18] Di Bono M. G. Zorzi M. (2008). Decoding cognitive states from fMRI data using support vector regression. Psychnol. J. 6, 189–201.

[B19] Ding X. Yang Y. Stein E. A. Ross T. J. (2017). Combining multiple resting-state fMRI features during classification: optimized frameworks and their application to nicotine addiction. Front. Hum. Neurosci. 11:362. 10.3389/fnhum.2017.00362 28747877 PMC5506584

[B20] Dosenbach N. U. Nardos B. Cohen A. L. Fair D. A. Power J. D. Church J. A.. (2010). Prediction of individual brain maturity using fMRI. Science 329, 1358–1361. 10.1126/science.1194144 20829489 PMC3135376

[B21] Felton E. Radwin R. Wilson J. Williams J. (2009). Evaluation of a modified Fitts law brain–computer interface target acquisition task in able and motor disabled individuals. J. Neural Eng. 6:056002. 10.1088/1741-2560/6/5/056002 19700814 PMC4075430

[B22] Fergus P. Hussain A. Hignett D. Al-Jumeily D. Abdel-Aziz K. Hamdan H. (2016). A machine learning system for automated whole-brain seizure detection. Appl. Comput. Inform. 12, 70–89. 10.1016/j.aci.2015.01.001

[B23] Gomez-Pilar J. Corralejo R. Nicolas-Alonso L. Álvarez D. Hornero R. (2014). Assessment of neurofeedback training by means of motor imagery based-bci for cognitive rehabilitation, Engineering in Medicine and Biology Society (EMBC), in 36th Annual International Conference of the IEEE, 2014 (Chicago, IL: IEEE), 3630–3633. 10.1109/EMBC.2014.6944409 25570777

[B24] Gordon N. F. Gulanick M. Costa F. Fletcher G. Franklin B. A. Roth E. J.. (2004). Physical activity and exercise recommendations for stroke survivors. Stroke 35, 1230–1240. 10.1161/01.STR.0000127303.19261.19 15105522

[B25] Grefkes C. Eickhoff S. B. Nowak D. A. Dafotakis M. Fink G. R. (2008). Dynamic intra-and interhemispheric interactions during unilateral and bilateral hand movements assessed with fMRI and DCM. Neuroimage 41, 1382–1394. 10.1016/j.neuroimage.2008.03.048 18486490

[B26] Hastie T. Tibshirani R. Friedman J. (2001). The Elements of Statistical Learning. New York, NY: Springer.

[B27] Hoffmann N. Koch E. Steiner G. Petersohn U. Kirsch M. (2016). Learning thermal process representations for intraoperative analysis of cortical perfusion during ischemic strokes, in Deep Learning and Data Labeling for Medical Applications (Athens: Springer), 152–160.

[B28] Jackson J. E. (2005). A User's Guide to Principal Components. New York, NY: John Wiley and Sons.

[B29] Jolliffe I. T. (1986) Principal Component Analysis. New York, NY: Springer-Verlag.

[B30] Kamnitsas K. Ledig C. Newcombe V. F. Simpson J. P. Kane A. D. Menon D. K.. (2017). Efficient multi-scale 3D CNN with fully connected CRF for accurate brain lesion segmentation. Med. Image Anal. 36, 61–78. 10.1016/j.media.2016.10.004 27865153

[B31] Khazaee A. Ebrahimzadeh A. Babajani-Feremi A. (2016). Application of advanced machine learning methods on resting-state fMRI network for identification of mild cognitive impairment and Alzheimer's disease. Brain Imaging Behav. 10, 799–817. 10.1007/s11682-015-9448-7 26363784

[B32] Kwakkel G. Kollen B. J. Krebs H. I. (2008). Effects of robot-assisted therapy on upper limb recovery after stroke: a systematic review. Neurorehabil. Neural Repair 22, 111–121. 10.1177/1545968307305457 17876068 PMC2730506

[B33] Lang C. E. Wagner J. M. Dromerick A. W. Edwards D. F. (2006). Measurement of upper-extremity function early after stroke: properties of the action research arm test. Arch. Phys. Med. Rehabil. 87, 1605–1610. 10.1016/j.apmr.2006.09.003 17141640

[B34] Lee M. H. Smyser C. D. Shimony J. S. (2013). Resting-state fMRI: a review of methods and clinical applications. Am. J. Neuroradiol. 34, 1866–1872. 10.3174/ajnr.A3263 22936095 PMC4035703

[B35] Leys C. Ley C. Klein O. Bernard P. Licata L. (2013). Detecting outliers: do not use standard deviation around the mean, use absolute deviation around the median. J. Exp. Soc. Psychol. 49, 764–766. 10.1016/j.jesp.2013.03.013

[B36] Lohse K. R. Hilderman C. G. Cheung K. L. Tatla S. Van der Loos H. M. (2014). Virtual reality therapy for adults post-stroke: a systematic review and meta-analysis exploring virtual environments and commercial games in therapy. PLoS ONE 9:e93318. 10.1371/journal.pone.0093318 24681826 PMC3969329

[B37] Lotte F. Congedo M. Lécuyer A. Lamarche F. Arnaldi B. (2007). A review of classification algorithms for EEG-based brain–computer interfaces. J. Neural Eng. 4:R1. 10.1088/1741-2560/4/2/R01 17409472

[B38] Lotze M. Montoya P. Erb M. Hülsmann E. Flor H. Klose U.. (1999). Activation of cortical and cerebellar motor areas during executed and imagined hand movements: an fMRI study. J. Cogn. Neurosci. 11, 491–501. 10.1162/089892999563553 10511638

[B39] Mason S. G. Birch G. E. (2000). A brain-controlled switch for asynchronous control applications. IEEE Trans. Biomed. Eng. 47, 1297–1307. 10.1109/10.871402 11059164

[B40] Massey F. J. Jr. (1951). The Kolmogorov-Smirnov test for goodness of fit. J. Am. Stat. Assoc. 46, 68–78. 10.1080/01621459.1951.10500769

[B41] Meier T. B. Desphande A. S. Vergun S. Nair V. A. Song J. Biswal B. B.. (2012). Support vector machine classification and characterization of age-related reorganization of functional brain networks. Neuroimage 60, 601–613. 10.1016/j.neuroimage.2011.12.052 22227886 PMC3288439

[B42] Mohanty R. Sinha A. Remsik A. Allen J. Nair V. Caldera K. (2017). Machine learning-based prediction of changes in behavioral outcomes using functional connectivity and clinical measures in brain-computer interface stroke, rehabilitation, in International Conference on Augmented Cognition (Vancoucer, BC: Springer), 543–557.

[B43] Mugler E. Flint R. Wright Z. Schuele S. Rosenow J. Patton J. (2013). Decoding articulatory properties of overt speech from electrocorticography, in Proceeding Fifth International Brain-Computer Interface Meet 2013 (Pacific Grove, CA), 4–5.

[B44] Muller K.-R. Anderson C. W. Birch G. E. (2003). Linear and nonlinear methods for brain-computer interfaces. IEEE Trans. Neural Syst. Rehabil. Eng. 11, 165–169. 10.1109/TNSRE.2003.814484 12899264

[B45] Murphy K. Fox M. D. (2016). Towards a consensus regarding global signal regression for resting state functional connectivity MRI. Neuroimage 154, 169–173. 10.1016/j.neuroimage.2016.11.052 27888059 PMC5489207

[B46] Nair V. A. Young B. M. Nigogosyan Z. Remsick A. Weber S. Diffee K. (2015). Resting-state functional connectivity changes after stroke rehabilitation using closed loop neurofeedback, International Stroke Conference (Nashville, TN: American Heart Association).

[B47] Neshige R. Murayama N. Igasaki T. Tanoue K. Kurokawa H. Asayama S. (2007). Communication aid device utilizing event-related potentials for patients with severe motor impairment. Brain Res. 1141, 218–227. 10.1016/j.brainres.2006.12.003 17320055

[B48] Noble W. S. (2006). What is a support vector machine? Nat. Biotechnol. 24, 1565–1567. 10.1038/nbt1206-1565 17160063

[B49] Payan A. Montana G. (2015). Predicting Alzheimer's disease: a neuroimaging study with 3D convolutional neural networks. *arXiv:150202506*.

[B50] Power J. D. Cohen A. L. Nelson S. M. Wig G. S. Barnes K. A. Church J. A.. (2011). Functional network organization of the human brain. Neuron 72, 665–678. 10.1016/j.neuron.2011.09.006 22099467 PMC3222858

[B51] Rakotomamonjy A. Guigue V. (2008). BCI competition III: dataset II-ensemble of SVMs for BCI P300 speller. IEEE Trans. Biomed. Eng. 55, 1147–1154. 10.1109/TBME.2008.915728 18334407

[B52] Rehme A. K. Volz L. J. Feis D.-L. Bomilcar-Focke I. Liebig T. Eickhoff S. B.. (2014). Identifying neuroimaging markers of motor disability in acute stroke by machine learning techniques. Cereb. cortex 25, 3046–3056. 10.1093/cercor/bhu100 24836690

[B53] Schalk G. McFarland D. J. Hinterberger T. Birbaumer N. Wolpaw J. R. (2004). BCI2000: a general-purpose brain-computer interface (BCI) system. IEEE Trans. Biomed. Eng. 51, 1034–1043. 10.1109/TBME.2004.827072 15188875

[B54] Selim A. Wahed M. A. Kadah Y. (2008). Machine learning methodologies in brain-computer interface systems, in Biomedical Engineering Conference 2008 (Cairo: Cairo International; IEEE), 1–5.

[B55] Silvoni S. Ramos-Murguialday A. Cavinato M. Volpato C. Cisotto G. Turolla A.. (2011). Brain-computer interface in stroke: a review of progress. Clin. EEG Neurosci. 42, 245–252. 10.1177/155005941104200410 22208122

[B56] Smith S. M. Miller K. L. Salimi-Khorshidi G. Webster M. Beckmann C. F. Nichols T. E.. (2011). Network modelling methods for FMRI. Neuroimage 54, 875–891. 10.1016/j.neuroimage.2010.08.063 20817103

[B57] Snoek J. Larochelle H. Adams R. P. (2012). Practical bayesian optimization of machine learning algorithms, in Advances in Neural Information Processing Systems (Lake Tahoe), 2012, 2951–2959.

[B58] Soekadar S. R. Birbaumer N. Slutzky M. W. Cohen L. G. (2015). Brain–machine interfaces in neurorehabilitation of stroke. Neurobiol. Dis. 83, 172–179. 10.1016/j.nbd.2014.11.025 25489973

[B59] Song J. Young B. M. Nigogosyan Z. Walton L. M. Nair V. A. Grogan S. W.. (2014). Characterizing relationships of DTI, fMRI, and motor recovery in stroke rehabilitation utilizing brain-computer interface technology. Front. Neuroeng. 7:31. 10.3389/fneng.2014.00031 25120466 PMC4114288

[B60] Sun H. Y. Xiang Y. , Yang, M. D. (2011). Neurological rehabilitation of stroke patients via motor imaginary-based braincomputer interface technology. Neural Regen. Res. 6, 2198–2202.

[B61] Takeuchi N. Izumi S.-I. (2012). Maladaptive plasticity for motor recovery after stroke: mechanisms and approaches. Neural Plast. 2012:359728. 10.1155/2012/359728 22792492 PMC3391905

[B62] Van Den Heuvel M. P. Pol H. E. H. (2010). Exploring the brain network: a review on resting-state fMRI functional connectivity. Eur. Neuropsychopharmacol. 20, 519–534. 10.1016/j.euroneuro.2010.03.008 20471808

[B63] Várkuti B. Guan C. Pan Y. Phua K. S. Ang K. K. Kuah C. W. K.. (2013). Resting state changes in functional connectivity correlate with movement recovery for BCI and robot-assisted upper-extremity training after stroke. Neurorehabil. Neural Repair 27, 53–62. 10.1177/1545968312445910 22645108

[B64] Vergun S. Deshpande A. S. Meier T. B. Song J. Tudorascu D. L. Nair V. A.. (2013). Characterizing functional connectivity differences in aging adults using machine learning on resting state fMRI data. Front. Comput. Neurosci. 7:38. 10.3389/fncom.2013.00038 23630491 PMC3635030

[B65] Wilson J. A. Schalk G. Walton L. M. Williams J. C. (2009). Using an EEG-based brain-computer interface for virtual cursor movement with BCI2000. J. Vis. Exp. 2009:e1319 10.3791/1319 PMC2900251 19641479

[B66] Wilson J. A. Walton L. M. Tyler M. Williams J. (2012). Lingual electrotactile stimulation as an alternative sensory feedback pathway for brain–computer interface applications. J. Neural Eng. 9:045007. 10.1088/1741-2560/9/4/045007 22832032

[B67] Xia M. Wang J. He Y. (2013). BrainNet viewer: a network visualization tool for human brain connectomics. PLoS ONE 8:e68910. 10.1371/journal.pone.0068910 23861951 PMC3701683

[B68] Young B. M. Nigogosyan Z. Nair V. A. Walton L. M. Song J. Tyler M. E.. (2014a). Case report: post-stroke interventional BCI rehabilitation in an individual with preexisting sensorineural disability. Front. Neuroeng. 7:18. 10.3389/fneng.2014.00018 25009491 PMC4067954

[B69] Young B. M. Nigogosyan Z. Walton L. M. Song J. Nair V. A. Grogan S. W.. (2014b). Changes in functional brain organization and behavioral correlations after rehabilitative therapy using a brain-computer interface. Front. Neuroeng. 7:26. 10.3389/fneng.2014.00026 25076886 PMC4097124

[B70] Zelen M. (1974). The randomization and stratification of patients to clinical trials. J. Chronic Dis. 27, 365–375. 10.1016/0021-9681(74)90015-0 4612056

