# Supplementary material for: Machine Learning Classification to Identify the Stage of Brain-Computer Interface Therapy for Stroke Rehabilitation Using Functional Connectivity
Source: Front Neurosci. 2018 May 29;12:353. doi: 10.3389/fnins.2018.00353 (PMC5986965; doi:10.3389/fnins.2018.00353)
Supplement: Supplementary file 1 [file Data_Sheet_1.PDF]

Supplementary Material

Machine Learning Classification to Identify the Stage of Brain-Computer Interface Therapy for Stroke Rehabilitation using Functional Connectivity

Rosaleena Mohanty*, Anita M. Sinha, Alexander Remsik, Keith Dodd, Tyler Jacobson, Matthew McMillan, Jaclyn Thoma, Hemali Advani, Brittany M. Young, Veena A. Nair, Theresa J. Kang, Kristin Caldera, Dorothy F. Edwards, Justin C. Williams, Vivek Prabhakaran

*** Correspondence:** Rosaleena Mohanty: rmohanty@wisc.edu

1 Supplementary Tables

Supplementary Table 1. Complete list of 441 strengthening connections that strengthened from pre- to post-therapy provides the pair of seed regions of interest (ROI) involved specified by the network they belong to and their anatomical label in MNI coordinates. The connections are arranged in descending order of contribution in classification.

ROI1 - Network	ROI1 - Label	ROI2 - Network	ROI2 - Label
Salience	R Superior Frontal Gyrus & R BA 10	Default mode	R Superior Frontal Gyrus
Default mode	R Inferior Frontal Gyrus	Default mode	R Pyramis
Ventral attention	L Superior Frontal Gyrus & L BA 6	Default mode	L Superior Frontal Gyrus
Visual	R Middle Occipital Gyrus & R BA 19	Default mode	R Pyramis
Subcortical	R Lentiform Nucleus & R Putamen	Default mode	R Anterior Cingulate & R BA 32
Default mode	L Precuneus	Fronto parietal Task Control	R Middle Frontal Gyrus
Sensory somatomotor Hand	L Precentral Gyrus	Default mode	L Medial Frontal Gyrus & L BA 10
Fronto parietal Task Control	L Inferior Frontal Gyrus	Default mode	R Medial Frontal Gyrus & R BA 9
Visual	R Declive	Default mode	R Medial Frontal Gyrus & R BA 9
Fronto parietal Task Control	L Inferior Frontal Gyrus	Default mode	L Superior Frontal Gyrus
Default mode	L Precuneus	Default mode	L Parahippocampal Gyrus & L BA 36
Auditory	L Superior Temporal Gyrus	Auditory	L Superior Temporal Gyrus & L BA 42

Subcortical	L Thalamus	Default mode	R Superior Frontal Gyrus
Sensory somatomotor Hand	L Postcentral Gyrus	Default mode	L Medial Frontal Gyrus & L BA 10
Default mode	L Middle Temporal Gyrus	Visual	L Lingual Gyrus
Visual	R Cuneus & R BA 17	Visual	R Cuneus & R BA 19
Dorsal attention	R Precuneus & R BA 7	Default mode	L Superior Frontal Gyrus
Subcortical	L Thalamus	Default mode	L Anterior Cingulate
Default mode	L Superior Occipital Gyrus	Fronto parietal Task Control	R Inferior Parietal Lobule
Subcortical	R Lentiform Nucleus & R Putamen	Visual	L Lingual Gyrus & L BA 19
Fronto parietal Task Control	L Middle Frontal Gyrus	Visual	R Middle Occipital Gyrus
Default mode	L Parahippocampal Gyrus & L BA 30	Subcortical	R Lentiform Nucleus & R Putamen
Dorsal attention	L Precuneus & L BA 19	Memory retrieval	R Precuneus
Visual	R Middle Occipital Gyrus	Default mode	R Pyramis
Fronto parietal Task Control	L Inferior Parietal Lobule & L BA 40	Default mode	L Parahippocampal Gyrus & L BA 36
Visual	L Fusiform Gyrus & L BA 19	Default mode	L Superior Frontal Gyrus
Cingulo opercular Task Control	L Claustrum	Default mode	R Inferior Frontal Gyrus
Fronto parietal Task Control	R Middle Frontal Gyrus & R BA 6	Default mode	L Medial Frontal Gyrus
Subcortical	R Lentiform Nucleus & R Putamen	Salience	R Middle Frontal Gyrus
Sensory somatomotor Hand	R Precentral Gyrus	Visual	L Cuneus & L BA 18
Memory retrieval	R Precuneus & R BA 7	Default mode	R Parahippocampal Gyrus
Subcortical	L Thalamus	Subcortical	R Thalamus
Default mode	L Posterior Cingulate	Default mode	L Inferior Frontal Gyrus & L BA 47
Auditory	R Insula & R BA 13	Memory retrieval	R Cingulate Gyrus
Cingulo opercular Task Control	R Superior Frontal Gyrus	Fronto parietal Task Control	L Middle Frontal Gyrus & L BA 6
Cerebellar	L Culmen	Default mode	L Middle Temporal Gyrus
Sensory somatomotor Hand	R Precentral Gyrus	Fronto parietal Task Control	R Middle Frontal Gyrus

Default mode	L Middle Temporal Gyrus	Dorsal attention	R Precuneus & R BA 7
Dorsal attention	Superior Parietal Lobule	Cerebellar	R Declive
Default mode	L Superior Occipital Gyrus	Auditory	R Precentral Gyrus & R BA 43
Default mode	R Precuneus & R BA 31	Subcortical	L Thalamus
Default mode	L Superior Frontal Gyrus & L BA 6	Subcortical	R Thalamus
Salience	L Superior Frontal Gyrus & L BA 10	Default mode	L Middle Temporal Gyrus
Salience	R Superior Frontal Gyrus	Visual	R Cuneus & R BA 19
Salience	L Middle Frontal Gyrus & L BA 10	Salience	L Insula & L BA 13
Sensory somatomotor Hand	R Postcentral Gyrus & R BA 5	Default mode	R Medial Frontal Gyrus & R BA 10
Subcortical	L Thalamus	Default mode	L Superior Frontal Gyrus
Salience	L Insula & L BA 13	Visual	L Precuneus & L BA 7
Sensory somatomotor Hand	L Precentral Gyrus & L BA 4	Subcortical	R Thalamus
Default mode	L Angular Gyrus & L BA 39	Visual	L Cuneus
Default mode	L Superior Frontal Gyrus	Cerebellar	L Culmen
Default mode	L Superior Temporal Gyrus & L BA 39	Default mode	L Anterior Cingulate
Subcortical	R Thalamus & R Ventral Anterior Nucleus	Fronto parietal Task Control	L Middle Frontal Gyrus & L BA 6
Default mode	L Middle Temporal Gyrus	Default mode	L Anterior Cingulate & L BA 10
Cingulo opercular Task Control	R Insula	Default mode	R Pyramis
Default mode	R Posterior Cingulate	Visual	R Cuneus & R BA 17
Sensory somatomotor Hand	Not Found	Visual	R Cuneus & R BA 19
Default mode	L Middle Temporal Gyrus	Fronto parietal Task Control	R Middle Frontal Gyrus & R BA 11
Default mode	R Precuneus & R BA 31	Cerebellar	L Culmen
Auditory	L Insula	Default mode	R Superior Frontal Gyrus
Default mode	L Middle Temporal	Visual	L Lingual Gyrus & L

	Gyrus		BA 19
Subcortical	R Lentiform Nucleus & R Putamen	Visual	R Cuneus & R BA 19
Cerebellar	L Culmen	Fronto parietal Task Control	R Inferior Parietal Lobule & R BA 40
Auditory	L Inferior Parietal Lobule	Default mode	R Middle Temporal Gyrus
Sensory somatomotor Hand	L Precentral Gyrus & L BA 4	Visual	R Lingual Gyrus
Dorsal attention	L Middle Temporal Gyrus	Ventral attention	L Superior Temporal Gyrus & L BA 22
Default mode	R Posterior Cingulate	Salience	R Middle Frontal Gyrus
Sensory somatomotor Hand	L Inferior Parietal Lobule & L BA 40	Cerebellar	R Declive
Subcortical	R Thalamus	Visual	L Lingual Gyrus & L BA 19
Default mode	L Superior Frontal Gyrus & L BA 8	Default mode	L Anterior Cingulate
Auditory	L Insula	Default mode	L Anterior Cingulate
Ventral attention	R Inferior Parietal Lobule & R BA 40	Visual	R Declive
Subcortical	L Lentiform Nucleus & L Putamen	Visual	R Cuneus
Dorsal attention	L Precuneus & L BA 19	Default mode	L Superior Frontal Gyrus
Visual	R Culmen	Default mode	R Anterior Cingulate & R BA 32
Visual	L Cuneus	Default mode	L Superior Frontal Gyrus
Default mode	L Precuneus	Visual	R Cuneus & R BA 19
Default mode	L Parahippocampal Gyrus & L BA 30	Salience	L Middle Frontal Gyrus & L BA 10
Default mode	L Superior Frontal Gyrus & L BA 8	Dorsal attention	R Precuneus & R BA 7
Sensory somatomotor Hand	Not Found	Visual	R Cuneus
Subcortical	R Thalamus	Visual	R Fusiform Gyrus
Default mode	L Angular Gyrus & L BA 39	Visual	L Lingual Gyrus & L BA 19
Fronto parietal Task Control	R Inferior Parietal Lobule	Default mode	L Medial Frontal Gyrus & L BA 6
Fronto parietal Task Control	R Inferior Parietal Lobule & R BA 40	Visual	R Declive
Subcortical	L Thalamus	Memory retrieval	L Cingulate Gyrus & L BA 31

Cingulo opercular Task Control	L Medial Frontal Gyrus & R BA 6	Default mode	R Anterior Cingulate & R BA 32
Visual	R Middle Occipital Gyrus	Default mode	R Medial Frontal Gyrus & R BA 9
Default mode	L Middle Temporal Gyrus	Visual	R Middle Occipital Gyrus
Cingulo opercular Task Control	R Insula	Fronto parietal Task Control	R Middle Frontal Gyrus
Fronto parietal Task Control	R Middle Frontal Gyrus & R BA 6	Default mode	L Superior Frontal Gyrus
Fronto parietal Task Control	R Inferior Temporal Gyrus & R BA 20	Memory retrieval	R Precuneus
Sensory somatomotor Hand	R Postcentral Gyrus & R BA 3	Fronto parietal Task Control	L Middle Frontal Gyrus & L BA 10
Auditory	L Precentral Gyrus & L BA 43	Fronto parietal Task Control	R Inferior Parietal Lobule
Ventral attention	R Inferior Parietal Lobule & R BA 40	Default mode	L Inferior Frontal Gyrus & L BA 47
Default mode	L Precuneus	Fronto parietal Task Control	L Superior Parietal Lobule & L BA 7
Visual	R Middle Occipital Gyrus	Memory retrieval	R Precuneus
Dorsal attention	R Precuneus & R BA 7	Fronto parietal Task Control	L Middle Frontal Gyrus
Subcortical	L Thalamus	Visual	L Middle Occipital Gyrus
Fronto parietal Task Control	R Inferior Parietal Lobule	Visual	R Declive
Fronto parietal Task Control	R Inferior Temporal Gyrus & R BA 20	Default mode	L Medial Frontal Gyrus & L BA 6
Default mode	L Middle Temporal Gyrus	Default mode	R Supramarginal Gyrus
Default mode	L Precuneus	Auditory	R Superior Temporal Gyrus & R BA 42
Fronto parietal Task Control	R Inferior Temporal Gyrus & R BA 20	Default mode	R Supramarginal Gyrus
Default mode	R Posterior Cingulate	Auditory	L Precentral Gyrus & L BA 43
Default mode	L Parahippocampal Gyrus & L BA 36	Default mode	R Anterior Cingulate & R BA 32
Default mode	L Middle Temporal Gyrus	Visual	L Middle Occipital Gyrus
Sensory somatomotor Hand	R Superior Frontal Gyrus	Subcortical	R Thalamus
Fronto parietal Task Control	R Middle Frontal Gyrus	Visual	R Declive
Auditory	L Precentral Gyrus &	Default mode	L Middle Temporal

	L BA 43		Gyrus
Default mode	L Middle Temporal Gyrus	Visual	L Fusiform Gyrus & L BA 19
Ventral attention	R Inferior Parietal Lobule & R BA 40	Visual	R Cuneus & R BA 19
Default mode	L Angular Gyrus & L BA 39	Subcortical	L Thalamus & L Medial Dorsal Nucleus
Visual	L Lingual Gyrus	Default mode	L Parahippocampal Gyrus & L BA 36
Dorsal attention	L Precuneus & L BA 19	Salience	R Superior Frontal Gyrus & R BA 10
Sensory somatomotor Hand	R Precentral Gyrus	Salience	L Insula & L BA 13
Auditory	L Postcentral Gyrus	Cingulo opercular Task Control	R Superior Frontal Gyrus
Fronto parietal Task Control	R Middle Frontal Gyrus	Default mode	R Pyramis
Default mode	L Parahippocampal Gyrus & L BA 30	Salience	R Insula & R BA 13
Subcortical	R Thalamus	Sensory somatomotor Hand	L Medial Frontal Gyrus
Sensory somatomotor Hand	R Precentral Gyrus	Fronto parietal Task Control	L Middle Frontal Gyrus
Subcortical	R Lentiform Nucleus & R Putamen	Fronto parietal Task Control	R Middle Frontal Gyrus
Fronto parietal Task Control	R Middle Frontal Gyrus	Default mode	R Superior Frontal Gyrus
Dorsal attention	R Precuneus & R BA 7	Default mode	R Parahippocampal Gyrus
Subcortical	R Thalamus	Default mode	R Medial Frontal Gyrus & R BA 9
Default mode	R Middle Temporal Gyrus & R BA 39	Default mode	R Inferior Temporal Gyrus
Subcortical	R Thalamus	Visual	L Fusiform Gyrus & L BA 19
Cingulo opercular Task Control	R Superior Frontal Gyrus	Subcortical	L Lentiform Nucleus & L Putamen
Fronto parietal Task Control	L Inferior Parietal Lobule	Visual	R Cuneus & R BA 17
Default mode	L Middle Temporal Gyrus	Default mode	R Medial Frontal Gyrus & R BA 10
Default mode	L Cingulate Gyrus & L BA 31	Visual	R Cuneus & R BA 19
Auditory	L Insula	Default mode	R Medial Frontal Gyrus & R BA 10

Default mode	R Posterior Cingulate	Default mode	L Middle Temporal Gyrus
Fronto parietal Task Control	L Middle Frontal Gyrus	Visual	R Cuneus & R BA 19
Auditory	L Insula	Subcortical	R Thalamus & R Ventral Anterior Nucleus
Visual	R Cuneus & R BA 23	Default mode	R Medial Frontal Gyrus & R BA 10
Auditory	R Postcentral Gyrus	Memory retrieval	L Precuneus & L BA 7
Default mode	L Superior Occipital Gyrus	Fronto parietal Task Control	L Inferior Parietal Lobule
Cingulo opercular Task Control	L Claustrum	Sensory somatomotor Hand	R Precentral Gyrus
Sensory somatomotor Hand	L Postcentral Gyrus	Default mode	L Parahippocampal Gyrus & L BA 36
Visual	L Middle Occipital Gyrus	Default mode	R Middle Temporal Gyrus
Auditory	L Superior Temporal Gyrus & L BA 42	Ventral attention	L Superior Temporal Gyrus
Fronto parietal Task Control	R Inferior Parietal Lobule	Visual	R Cuneus & R BA 19
Auditory	L Superior Temporal Gyrus & L BA 42	Default mode	L Middle Temporal Gyrus
Ventral attention	L Inferior Frontal Gyrus & L BA 47	Salience	L Insula & L BA 13
Default mode	L Middle Temporal Gyrus	Fronto parietal Task Control	R Inferior Temporal Gyrus & R BA 20
Sensory somatomotor Hand	R Precentral Gyrus	Visual	R Cuneus & R BA 23
Fronto parietal Task Control	L Middle Frontal Gyrus	Fronto parietal Task Control	R Inferior Parietal Lobule
Default mode	L Superior Frontal Gyrus & L BA 8	Default mode	L Parahippocampal Gyrus & L BA 30
Subcortical	R Lentiform Nucleus & R Putamen	Visual	R Cuneus & R BA 17
Cingulo opercular Task Control	R Medial Frontal Gyrus	Visual	L Lingual Gyrus
Default mode	L Parahippocampal Gyrus & L BA 30	Salience	L Superior Frontal Gyrus & L BA 10
Visual	R Cuneus & R BA 19	Default mode	R Anterior Cingulate & R BA 32
Default mode	L Superior Frontal Gyrus	Auditory	R Superior Temporal Gyrus & R BA 42
Default mode	L Middle Temporal Gyrus	Default mode	R Middle Temporal Gyrus

Cerebellar	L Declive	Visual	R Cuneus & R BA 19
Cingulo opercular Task Control	R Superior Temporal Gyrus	Default mode	R Anterior Cingulate & R BA 32
Fronto parietal Task Control	R Middle Frontal Gyrus	Visual	R Cuneus
Subcortical	L Lentiform Nucleus & L Putamen	Visual	R Fusiform Gyrus
Default mode	L Superior Frontal Gyrus & L BA 6	Fronto parietal Task Control	L Inferior Frontal Gyrus
Sensory somatomotor Hand	L Inferior Parietal Lobule & L BA 40	Fronto parietal Task Control	L Middle Frontal Gyrus
Fronto parietal Task Control	R Middle Frontal Gyrus	Visual	R Cuneus & R BA 19
Sensory somatomotor Hand	R Precentral Gyrus	Default mode	L Anterior Cingulate
Fronto parietal Task Control	L Middle Frontal Gyrus	Visual	R Middle Occipital Gyrus & R BA 19
Dorsal attention	R Middle Frontal Gyrus	Salience	R Superior Frontal Gyrus & R BA 10
Dorsal attention	L Precuneus & L BA 19	Fronto parietal Task Control	L Middle Frontal Gyrus
Default mode	L Superior Frontal Gyrus & L BA 6	Visual	L Cuneus
Default mode	R Pyramis	Default mode	R Superior Frontal Gyrus
Sensory somatomotor Mouth	R Precentral Gyrus	Default mode	L Medial Frontal Gyrus
Sensory somatomotor Hand	R Postcentral Gyrus & R BA 3	Visual	R Culmen
Salience	L Insula & L BA 13	Visual	R Cuneus & R BA 19
Default mode	L Superior Frontal Gyrus & L BA 6	Sensory somatomotor Hand	R Precentral Gyrus
Sensory somatomotor Hand	R Postcentral Gyrus & R BA 5	Visual	R Culmen
Subcortical	L Lentiform Nucleus & L Putamen	Salience	R Paracentral Lobule
Default mode	L Angular Gyrus & L BA 39	Visual	L Cuneus
Subcortical	R Lentiform Nucleus & R Putamen	Visual	R Culmen
Default mode	L Superior Frontal Gyrus & L BA 6	Sensory somatomotor Hand	L Postcentral Gyrus
Default mode	L Superior Frontal Gyrus	Subcortical	L Lentiform Nucleus & L Putamen
Cingulo opercular Task Control	R Medial Frontal Gyrus	Subcortical	R Thalamus & R Ventral Anterior

			Nucleus
Default mode	L Superior Occipital Gyrus	Fronto parietal Task Control	R Middle Frontal Gyrus
Sensory somatomotor Hand	R Postcentral Gyrus	Fronto parietal Task Control	L Middle Frontal Gyrus & L BA 10
Cingulo opercular Task Control	R Medial Frontal Gyrus	Default mode	L Parahippocampal Gyrus & L BA 36
Auditory	R Superior Temporal Gyrus & R BA 42	Subcortical	R Thalamus & R Ventral Anterior Nucleus
Auditory	L Insula	Cingulo opercular Task Control	L Precentral Gyrus & L BA 44
Fronto parietal Task Control	L Inferior Parietal Lobule	Visual	L Fusiform Gyrus & L BA 19
Sensory somatomotor Mouth	R Precentral Gyrus & R BA 6	Visual	R Lingual Gyrus
Fronto parietal Task Control	R Middle Frontal Gyrus	Default mode	L Superior Frontal Gyrus
Fronto parietal Task Control	R Inferior Parietal Lobule & R BA 40	Default mode	L Inferior Frontal Gyrus & L BA 47
Default mode	R Middle Temporal Gyrus & R BA 39	Fronto parietal Task Control	L Middle Frontal Gyrus & L BA 10
Default mode	R Posterior Cingulate	Fronto parietal Task Control	L Inferior Parietal Lobule
Cingulo opercular Task Control	L Cingulate Gyrus	Visual	R Lingual Gyrus
Dorsal attention	L Inferior Parietal Lobule	Subcortical	L Thalamus
Cingulo opercular Task Control	L Superior Temporal Gyrus & L BA 22	Default mode	R Superior Frontal Gyrus
Subcortical	L Thalamus	Visual	L Lingual Gyrus
Sensory somatomotor Hand	L Paracentral Lobule	Default mode	L Anterior Cingulate
Cingulo opercular Task Control	R Insula	Default mode	R Parahippocampal Gyrus
Default mode	L Inferior Frontal Gyrus & L BA 47	Default mode	R Supramarginal Gyrus
Default mode	L Posterior Cingulate	Fronto parietal Task Control	R Inferior Parietal Lobule & R BA 40
Dorsal attention	R Superior Parietal Lobule & R BA 7	Default mode	R Medial Frontal Gyrus & R BA 10
Auditory	L Postcentral Gyrus	Salience	R Paracentral Lobule
Subcortical	L Lentiform Nucleus & L Putamen	Fronto parietal Task Control	R Middle Frontal Gyrus & R BA 6
Subcortical	L Thalamus	Default mode	L Medial Frontal Gyrus & L BA 6
Ventral attention	L Superior Temporal	Visual	L Middle Occipital

	Gyrus & L BA 22		Gyrus & L BA 19
Fronto parietal Task Control	L Middle Frontal Gyrus	Fronto parietal Task Control	L Superior Parietal Lobule & L BA 7
Auditory	R Precentral Gyrus & R BA 43	Visual	L Fusiform Gyrus & L BA 19
Auditory	L Insula	Default mode	L Anterior Cingulate & L BA 10
Auditory	R Insula & R BA 13	Visual	R Middle Occipital Gyrus
Salience	R Middle Frontal Gyrus	Visual	R Cuneus & R BA 19
Dorsal attention	L Inferior Parietal Lobule	Default mode	L Middle Temporal Gyrus
Default mode	R Precuneus & R BA 31	Fronto parietal Task Control	L Inferior Frontal Gyrus & L BA 9
Default mode	L Parahippocampal Gyrus & L BA 30	Fronto parietal Task Control	L Middle Frontal Gyrus
Visual	L Cuneus	Visual	R Cuneus & R BA 23
Default mode	L Precuneus	Visual	L Lingual Gyrus
Sensory somatomotor Hand	L Precentral Gyrus	Default mode	L Anterior Cingulate & L BA 10
Default mode	R Posterior Cingulate	Auditory	L Superior Temporal Gyrus & L BA 41
Fronto parietal Task Control	R Middle Frontal Gyrus	Visual	R Middle Occipital Gyrus
Default mode	R Precuneus & R BA 31	Auditory	L Precentral Gyrus & L BA 43
Fronto parietal Task Control	R Middle Frontal Gyrus	Visual	R Superior Occipital Gyrus
Fronto parietal Task Control	R Middle Frontal Gyrus	Visual	R Cuneus & R BA 23
Auditory	L Insula	Default mode	R Medial Frontal Gyrus & R BA 9
Sensory somatomotor Hand	R Postcentral Gyrus	Fronto parietal Task Control	L Middle Frontal Gyrus
Ventral attention	R Inferior Parietal Lobule & R BA 40	Fronto parietal Task Control	R Inferior Temporal Gyrus & R BA 20
Default mode	R Precuneus & R BA 31	Default mode	L Inferior Frontal Gyrus & L BA 47
Sensory somatomotor Hand	R Postcentral Gyrus	Visual	R Middle Occipital Gyrus
Cingulo opercular Task Control	R Superior Frontal Gyrus	Default mode	R Superior Frontal Gyrus
Sensory somatomotor Hand	R Superior Frontal Gyrus	Fronto parietal Task Control	L Inferior Frontal Gyrus
Sensory somatomotor	Not Found	Visual	L Middle Occipital

Hand			Gyrus & L BA 19
Fronto parietal Task Control	R Middle Frontal Gyrus	Default mode	R Medial Frontal Gyrus & R BA 9
Cingulo opercular Task Control	R Insula	Salience	L Cingulate Gyrus
Sensory somatomotor Hand	Not Found	Sensory somatomotor Hand	L Medial Frontal Gyrus
Salience	R Superior Frontal Gyrus & R BA 10	Fronto parietal Task Control	L Middle Frontal Gyrus & L BA 10
Subcortical	L Lentiform Nucleus & L Putamen	Fronto parietal Task Control	R Middle Frontal Gyrus
Subcortical	L Thalamus & L Medial Dorsal Nucleus	Fronto parietal Task Control	R Inferior Parietal Lobule
Default mode	R Middle Temporal Gyrus & R BA 21	Default mode	R Middle Temporal Gyrus
Salience	R Cingulate Gyrus	Default mode	R Superior Frontal Gyrus
Default mode	R Posterior Cingulate	Fronto parietal Task Control	R Middle Frontal Gyrus
Subcortical	L Lentiform Nucleus & L Putamen	Fronto parietal Task Control	L Middle Frontal Gyrus
Sensory somatomotor Hand	R Postcentral Gyrus	Fronto parietal Task Control	L Middle Frontal Gyrus
Memory retrieval	R Cingulate Gyrus	Visual	L Cuneus
Default mode	R Posterior Cingulate	Fronto parietal Task Control	L Inferior Frontal Gyrus & L BA 9
Fronto parietal Task Control	L Middle Frontal Gyrus & L BA 6	Default mode	L Superior Frontal Gyrus
Fronto parietal Task Control	L Middle Frontal Gyrus	Fronto parietal Task Control	L Inferior Frontal Gyrus & L BA 9
Visual	R Middle Occipital Gyrus	Default mode	L Superior Frontal Gyrus
Visual	R Middle Occipital Gyrus	Default mode	R Superior Frontal Gyrus
Sensory somatomotor Hand	L Postcentral Gyrus	Ventral attention	L Superior Temporal Gyrus & L BA 22
Fronto parietal Task Control	R Inferior Temporal Gyrus & R BA 20	Memory retrieval	L Precuneus & L BA 7
Visual	R Declive	Default mode	R Superior Frontal Gyrus
Sensory somatomotor Hand	Not Found	Visual	R Cuneus
Subcortical	R Lentiform Nucleus & R Putamen	Fronto parietal Task Control	R Middle Frontal Gyrus
Sensory somatomotor Hand	R Precentral Gyrus & R BA 4	Default mode	L Inferior Frontal Gyrus & L BA 47

Default mode	R Precuneus & R BA 31	Visual	R Declive
Default mode	L Parahippocampal Gyrus & L BA 30	Subcortical	L Lentiform Nucleus & L Putamen
Visual	R Cuneus & R BA 19	Default mode	L Superior Frontal Gyrus
Default mode	R Precuneus & R BA 31	Dorsal attention	R Middle Frontal Gyrus
Sensory somatomotor Hand	R Postcentral Gyrus & R BA 3	Visual	R Declive
Default mode	L Superior Frontal Gyrus & L BA 6	Visual	R Cuneus & R BA 23
Visual	R Cuneus & R BA 23	Visual	R Culmen
Sensory somatomotor Hand	L Postcentral Gyrus	Default mode	R Anterior Cingulate & R BA 32
Fronto parietal Task Control	L Middle Frontal Gyrus & L BA 10	Visual	R Middle Occipital Gyrus
Visual	L Inferior Occipital Gyrus & L BA 18	Default mode	L Middle Temporal Gyrus
Cingulo opercular Task Control	R Superior Frontal Gyrus & R BA 6	Fronto parietal Task Control	L Middle Frontal Gyrus & L BA 46
Cingulo opercular Task Control	R Superior Frontal Gyrus	Cingulo opercular Task Control	L Superior Frontal Gyrus
Sensory somatomotor Mouth	L Precentral Gyrus	Visual	R Middle Occipital Gyrus
Sensory somatomotor Hand	L Precentral Gyrus	Default mode	R Medial Frontal Gyrus & R BA 10
Visual	L Middle Occipital Gyrus	Default mode	L Middle Temporal Gyrus
Visual	R Lingual Gyrus	Default mode	L Anterior Cingulate
Sensory somatomotor Hand	L Postcentral Gyrus	Default mode	R Medial Frontal Gyrus & R BA 9
Default mode	L Superior Temporal Gyrus & L BA 39	Default mode	R Medial Frontal Gyrus & R BA 10
Sensory somatomotor Hand	L Paracentral Lobule	Fronto parietal Task Control	L Middle Frontal Gyrus & L BA 6
Default mode	R Precuneus & R BA 31	Visual	R Middle Occipital Gyrus
Sensory somatomotor Hand	R Postcentral Gyrus	Visual	R Culmen
Visual	R Cuneus	Default mode	R Medial Frontal Gyrus & R BA 10
Default mode	L Angular Gyrus & L BA 39	Visual	L Lingual Gyrus
Default mode	R Medial Frontal Gyrus	Subcortical	R Thalamus

Cingulo opercular Task Control	R Medial Frontal Gyrus	Default mode	L Fusiform Gyrus & L BA 20
Auditory	R Insula & R BA 13	Salience	R Cingulate Gyrus
Visual	R Cuneus & R BA 17	Visual	R Lingual Gyrus
Visual	L Cuneus	Default mode	L Parahippocampal Gyrus & L BA 36
Fronto parietal Task Control	L Inferior Parietal Lobule & L BA 40	Visual	R Cuneus & R BA 17
Dorsal attention	L Middle Temporal Gyrus	Ventral attention	R Inferior Parietal Lobule & R BA 40
Sensory somatomotor Hand	L Postcentral Gyrus	Default mode	L Anterior Cingulate & L BA 10
Default mode	L Superior Frontal Gyrus & L BA 6	Sensory somatomotor Hand	L Paracentral Lobule
Default mode	R Precuneus & R BA 31	Fronto parietal Task Control	R Inferior Temporal Gyrus & R BA 20
Fronto parietal Task Control	R Inferior Parietal Lobule & R BA 40	Visual	R Lingual Gyrus
Sensory somatomotor Hand	L Precentral Gyrus	Default mode	L Superior Frontal Gyrus
Fronto parietal Task Control	R Middle Frontal Gyrus	Memory retrieval	R Precuneus & R BA 7
Sensory somatomotor Hand	L Precentral Gyrus & L BA 4	Sensory somatomotor Hand	R Precentral Gyrus
Default mode	L Superior Occipital Gyrus	Memory retrieval	R Precuneus & R BA 7
Default mode	L Middle Temporal Gyrus	Default mode	R Medial Frontal Gyrus & R BA 10
Default mode	R Precuneus & R BA 31	Cingulo opercular Task Control	L Cingulate Gyrus
Auditory	R Postcentral Gyrus	Visual	L Lingual Gyrus
Fronto parietal Task Control	L Middle Frontal Gyrus	Default mode	R Supramarginal Gyrus
Cerebellar	L Culmen	Visual	L Cuneus
Sensory somatomotor Hand	L Precentral Gyrus	Default mode	L Anterior Cingulate
Default mode	R Precuneus & R BA 31	Fronto parietal Task Control	R Middle Frontal Gyrus & R BA 6
Fronto parietal Task Control	R Inferior Parietal Lobule & R BA 40	Visual	R Cuneus
Auditory	R Postcentral Gyrus	Salience	R Middle Frontal Gyrus & R BA 6
Dorsal attention	R Superior Parietal Lobule & R BA 7	Default mode	L Medial Frontal Gyrus
Default mode	L Parahippocampal Gyrus & L BA 36	Default mode	L Medial Frontal Gyrus & L BA 9
Visual	R Lingual Gyrus	Visual	L Lingual Gyrus

Sensory somatomotor Hand	Not Found	Default mode	L Middle Temporal Gyrus
Default mode	L Superior Frontal Gyrus & L BA 6	Dorsal attention	Superior Parietal Lobule
Default mode	R Superior Frontal Gyrus & R BA 8	Fronto parietal Task Control	R Middle Frontal Gyrus & R BA 6
Salience	L Middle Frontal Gyrus & L BA 10	Default mode	R Middle Temporal Gyrus & R BA 21
Auditory	R Superior Temporal Gyrus & R BA 42	Sensory somatomotor Hand	L Precentral Gyrus
Sensory somatomotor Mouth	R Precentral Gyrus	Fronto parietal Task Control	L Inferior Parietal Lobule
Cingulo opercular Task Control	R Postcentral Gyrus & R BA 2	Sensory somatomotor Hand	L Cingulate Gyrus
Sensory somatomotor Hand	R Precentral Gyrus	Visual	L Middle Occipital Gyrus & L BA 19
Default mode	L Superior Frontal Gyrus & L BA 6	Cingulo opercular Task Control	R Superior Frontal Gyrus
Subcortical	R Lentiform Nucleus & R Putamen	Sensory somatomotor Hand	L Precuneus
Auditory	R Insula & R BA 13	Visual	R Cuneus & R BA 23
Sensory somatomotor Hand	L Postcentral Gyrus	Sensory somatomotor Hand	R Precentral Gyrus
Auditory	R Precentral Gyrus & R BA 43	Visual	L Middle Occipital Gyrus & L BA 19
Fronto parietal Task Control	R Middle Frontal Gyrus	Default mode	R Medial Frontal Gyrus & R BA 10
Fronto parietal Task Control	R Middle Frontal Gyrus	Default mode	L Medial Frontal Gyrus & L BA 6
Sensory somatomotor Hand	R Precentral Gyrus & R BA 4	Visual	R Lingual Gyrus
Sensory somatomotor Hand	Not Found	Visual	R Lingual Gyrus
Salience	L Superior Frontal Gyrus & L BA 10	Fronto parietal Task Control	L Superior Parietal Lobule & L BA 7
Sensory somatomotor Hand	L Precentral Gyrus & L BA 4	Visual	R Lingual Gyrus
Sensory somatomotor Hand	L Precentral Gyrus	Visual	R Lingual Gyrus
Auditory	L Superior Temporal Gyrus	Sensory somatomotor Hand	L Cingulate Gyrus
Cingulo opercular Task Control	L Medial Frontal Gyrus & R BA 6	Visual	L Lingual Gyrus
Sensory somatomotor Hand	L Postcentral Gyrus	Default mode	R Medial Frontal Gyrus & R BA 10
Sensory somatomotor	R Postcentral Gyrus	Salience	R Middle Frontal

Hand			Gyrus & R BA 6
Default mode	L Superior Frontal Gyrus & L BA 8	Fronto parietal Task Control	R Inferior Parietal Lobule
Sensory somatomotor Mouth	L Precentral Gyrus	Visual	R Lingual Gyrus
Fronto parietal Task Control	R Inferior Parietal Lobule	Visual	R Cuneus
Sensory somatomotor Hand	L Paracentral Lobule	Visual	R Cuneus & R BA 19
Sensory somatomotor Hand	L Postcentral Gyrus	Sensory somatomotor Hand	R Postcentral Gyrus & R BA 5
Cerebellar	R Declive	Default mode	R Inferior Temporal Gyrus
Visual	R Middle Occipital Gyrus	Default mode	R Anterior Cingulate & R BA 32
Sensory somatomotor Hand	R Precentral Gyrus	Sensory somatomotor Hand	L Medial Frontal Gyrus
Cingulo opercular Task Control	L Cingulate Gyrus	Sensory somatomotor Hand	L Precentral Gyrus
Default mode	L Precuneus	Salience	L Superior Frontal Gyrus & L BA 10
Default mode	L Superior Frontal Gyrus & L BA 8	Dorsal attention	R Superior Parietal Lobule & R BA 7
Sensory somatomotor Hand	R Cingulate Gyrus & R BA 24	Visual	R Cuneus & R BA 19
Default mode	L Precuneus	Subcortical	L Thalamus & L Medial Dorsal Nucleus
Auditory	R Precentral Gyrus & R BA 43	Cingulo opercular Task Control	L Cingulate Gyrus
Default mode	R Angular Gyrus	Dorsal attention	R Middle Frontal Gyrus
Auditory	R Superior Temporal Gyrus	Visual	L Cuneus & L BA 18
Sensory somatomotor Hand	R Postcentral Gyrus & R BA 3	Visual	R Middle Occipital Gyrus & R BA 19
Auditory	R Precentral Gyrus & R BA 43	Visual	L Inferior Occipital Gyrus & L BA 18
Sensory somatomotor Hand	R Precentral Gyrus	Ventral attention	L Superior Frontal Gyrus & L BA 6
Default mode	R Precuneus & R BA 31	Cingulo opercular Task Control	L Medial Frontal Gyrus & R BA 6
Auditory	R Superior Temporal Gyrus	Fronto parietal Task Control	R Inferior Temporal Gyrus & R BA 20
Visual	L Lingual Gyrus	Default mode	L Middle Temporal Gyrus
Subcortical	R Thalamus	Default mode	L Superior Frontal

			Gyrus
Sensory somatomotor Hand	Not Found	Default mode	R Parahippocampal Gyrus
Subcortical	L Thalamus	Fronto parietal Task Control	R Inferior Parietal Lobule
Default mode	L Parahippocampal Gyrus & L BA 30	Salience	L Anterior Cingulate
Default mode	L Superior Frontal Gyrus & L BA 6	Dorsal attention	R Superior Parietal Lobule & R BA 7
Dorsal attention	R Middle Frontal Gyrus	Visual	R Lingual Gyrus
Sensory somatomotor Hand	R Precentral Gyrus	Visual	R Cuneus & R BA 19
Visual	R Lingual Gyrus	Default mode	L Superior Frontal Gyrus
Ventral attention	L Inferior Frontal Gyrus & L BA 47	Default mode	R Medial Frontal Gyrus & R BA 10
Dorsal attention	Superior Parietal Lobule	Sensory somatomotor Hand	L Medial Frontal Gyrus
Auditory	R Precentral Gyrus & R BA 43	Salience	L Medial Frontal Gyrus & L BA 6
Sensory somatomotor Hand	L Precentral Gyrus	Visual	R Lingual Gyrus
Ventral attention	R Superior Temporal Gyrus	Visual	L Cuneus & L BA 18
Salience	L Anterior Cingulate	Visual	L Lingual Gyrus & L BA 19
Ventral attention	L Superior Temporal Gyrus & L BA 22	Visual	L Middle Occipital Gyrus & L BA 19
Visual	L Middle Occipital Gyrus	Default mode	L Anterior Cingulate
Visual	R Precuneus & R BA 31	Default mode	R Middle Temporal Gyrus
Dorsal attention	R Middle Frontal Gyrus	Default mode	L Inferior Frontal Gyrus & L BA 47
Fronto parietal Task Control	R Middle Frontal Gyrus	Default mode	R Superior Frontal Gyrus & R BA 8
Default mode	L Middle Temporal Gyrus	Subcortical	R Lentiform Nucleus & R Putamen
Sensory somatomotor Hand	R Postcentral Gyrus & R BA 5	Default mode	L Middle Temporal Gyrus
Salience	R Supramarginal Gyrus	Fronto parietal Task Control	R Middle Frontal Gyrus & R BA 6
Sensory somatomotor Hand	R Superior Frontal Gyrus	Subcortical	R Lentiform Nucleus & R Putamen
Default mode	R Posterior Cingulate	Fronto parietal Task	L Inferior Frontal

		Control	Gyrus
Fronto parietal Task Control	L Middle Frontal Gyrus & L BA 9	Visual	L Lingual Gyrus
Subcortical	R Lentiform Nucleus & R Putamen	Visual	L Cuneus & L BA 18
Dorsal attention	R Middle Frontal Gyrus	Sensory somatomotor Hand	L Precentral Gyrus & L BA 4
Default mode	R Precuneus & R BA 31	Sensory somatomotor Hand	L Postcentral Gyrus
Cingulo opercular Task Control	R Insula	Salience	R Cingulate Gyrus & R BA 32
Visual	L Lingual Gyrus	Default mode	R Parahippocampal Gyrus
Visual	R Cuneus & R BA 17	Visual	R Culmen
Sensory somatomotor Hand	R Precentral Gyrus	Visual	R Lingual Gyrus
Visual	L Middle Occipital Gyrus & L BA 19	Default mode	L Superior Frontal Gyrus
Auditory	R Insula & R BA 13	Visual	L Lingual Gyrus & L BA 19
Visual	L Middle Occipital Gyrus & L BA 19	Default mode	L Anterior Cingulate
Default mode	L Cingulate Gyrus & L BA 31	Salience	L Superior Frontal Gyrus & L BA 10
Auditory	R Precentral Gyrus & R BA 43	Visual	L Middle Occipital Gyrus
Default mode	L Superior Occipital Gyrus	Salience	L Insula & L BA 13
Fronto parietal Task Control	R Middle Frontal Gyrus	Visual	L Inferior Occipital Gyrus & L BA 18
Fronto parietal Task Control	R Middle Frontal Gyrus	Default mode	L Fusiform Gyrus & L BA 20
Sensory somatomotor Hand	R Paracentral Lobule	Dorsal attention	Superior Parietal Lobule
Auditory	R Insula & R BA 13	Visual	L Cuneus
Sensory somatomotor Mouth	R Insula & R BA 13	Default mode	R Medial Frontal Gyrus & R BA 9
Salience	L Middle Frontal Gyrus & L BA 10	Fronto parietal Task Control	R Middle Frontal Gyrus & R BA 6
Ventral attention	R Inferior Parietal Lobule & R BA 40	Visual	R Middle Occipital Gyrus
Cingulo opercular Task Control	L Superior Frontal Gyrus	Dorsal attention	R Middle Frontal Gyrus
Cingulo opercular Task Control	R Insula	Default mode	L Anterior Cingulate
Sensory somatomotor Hand	R Postcentral Gyrus	Visual	R Superior Occipital Gyrus

Sensory somatomotor Mouth	L Postcentral Gyrus	Ventral attention	R Inferior Parietal Lobule & R BA 40
Sensory somatomotor Hand	R Postcentral Gyrus	Default mode	L Middle Temporal Gyrus
Default mode	L Superior Frontal Gyrus & L BA 6	Cingulo opercular Task Control	R Medial Frontal Gyrus
Ventral attention	R Inferior Parietal Lobule & R BA 40	Visual	L Middle Occipital Gyrus & L BA 19
Default mode	R Medial Frontal Gyrus	Subcortical	L Thalamus & L Medial Dorsal Nucleus
Default mode	L Angular Gyrus & L BA 39	Visual	R Lingual Gyrus
Dorsal attention	L Middle Temporal Gyrus	Visual	L Fusiform Gyrus & L BA 19
Auditory	R Insula & R BA 13	Subcortical	L Lentiform Nucleus & L Putamen
Fronto parietal Task Control	R Inferior Temporal Gyrus & R BA 20	Fronto parietal Task Control	L Inferior Parietal Lobule
Subcortical	L Thalamus & L Medial Dorsal Nucleus	Fronto parietal Task Control	R Inferior Parietal Lobule & R BA 40
Ventral attention	L Superior Temporal Gyrus & L BA 22	Default mode	R Medial Frontal Gyrus & R BA 10
Fronto parietal Task Control	R Middle Frontal Gyrus	Visual	L Middle Occipital Gyrus & L BA 19
Sensory somatomotor Hand	Not Found	Sensory somatomotor Hand	R Postcentral Gyrus & R BA 5
Cingulo opercular Task Control	L Cingulate Gyrus	Sensory somatomotor Hand	L Precentral Gyrus
Sensory somatomotor Hand	R Postcentral Gyrus	Visual	R Middle Occipital Gyrus
Visual	L Lingual Gyrus	Default mode	L Fusiform Gyrus & L BA 20
Sensory somatomotor Hand	R Paracentral Lobule	Visual	R Lingual Gyrus
Sensory somatomotor Hand	Not Found	Visual	L Cuneus
Visual	R Culmen	Default mode	L Middle Temporal Gyrus
Cingulo opercular Task Control	L Cingulate Gyrus	Sensory somatomotor Hand	L Postcentral Gyrus
Fronto parietal Task Control	R Inferior Parietal Lobule	Visual	R Fusiform Gyrus
Default mode	L Posterior Cingulate	Salience	L Superior Frontal Gyrus & L BA 10

Cingulo opercular Task Control	L Cingulate Gyrus	Sensory somatomotor Hand	L Precentral Gyrus & L BA 4
Cingulo opercular Task Control	L Cingulate Gyrus	Sensory somatomotor Hand	R Postcentral Gyrus & R BA 5
Fronto parietal Task Control	L Middle Frontal Gyrus	Visual	R Cuneus & R BA 17
Sensory somatomotor Hand	R Postcentral Gyrus	Default mode	L Middle Temporal Gyrus
Default mode	L Superior Frontal Gyrus & L BA 6	Visual	R Middle Occipital Gyrus
Cingulo opercular Task Control	L Cingulate Gyrus	Sensory somatomotor Hand	L Inferior Parietal Lobule & L BA 40
Sensory somatomotor Hand	R Paracentral Lobule	Visual	L Lingual Gyrus
Salience	R Supramarginal Gyrus	Fronto parietal Task Control	L Middle Frontal Gyrus
Ventral attention	L Superior Temporal Gyrus	Default mode	R Medial Frontal Gyrus & R BA 10
Sensory somatomotor Hand	R Postcentral Gyrus & R BA 5	Default mode	L Middle Temporal Gyrus
Visual	R Lingual Gyrus	Memory retrieval	L Cingulate Gyrus & L BA 31
Dorsal attention	R Precuneus & R BA 7	Visual	L Precuneus & L BA 7
Sensory somatomotor Hand	R Postcentral Gyrus & R BA 3	Visual	R Lingual Gyrus
Sensory somatomotor Hand	L Postcentral Gyrus	Memory retrieval	R Precuneus
Sensory somatomotor Hand	R Precentral Gyrus	Fronto parietal Task Control	L Middle Frontal Gyrus & L BA 6
Fronto parietal Task Control	R Middle Frontal Gyrus	Default mode	R Medial Frontal Gyrus & R BA 10
Fronto parietal Task Control	L Middle Frontal Gyrus & L BA 6	Memory retrieval	R Precuneus & R BA 7
Sensory somatomotor Mouth	R Precentral Gyrus & R BA 6	Sensory somatomotor Hand	R Precentral Gyrus
Sensory somatomotor Hand	R Cingulate Gyrus & R BA 24	Default mode	L Middle Temporal Gyrus
Auditory	R Precentral Gyrus & R BA 43	Salience	R Cingulate Gyrus & R BA 32
Visual	R Cuneus & R BA 17	Default mode	L Superior Frontal Gyrus
Cingulo opercular Task Control	L Cingulate Gyrus	Cingulo opercular Task Control	L Medial Frontal Gyrus & R BA 6
Default mode	R Superior Frontal Gyrus	Default mode	L Medial Frontal Gyrus & L BA 10
Cingulo opercular	L Cingulate Gyrus	Sensory somatomotor	L Postcentral Gyrus

Task Control		Hand	
Auditory	R Superior Temporal Gyrus & R BA 42	Cingulo opercular Task Control	L Superior Temporal Gyrus & L BA 22
Cingulo opercular Task Control	L Cingulate Gyrus	Sensory somatomotor Hand	R Postcentral Gyrus

Supplementary Table 2. Complete list of 238 weakening connections that weakened from pre- to post-therapy provides the pair of seed regions of interest (ROI) involved specified by the network they belong to and their anatomical label in MNI coordinates. The connections are arranged in descending order of their contribution in classification.

ROI1 - Network	ROI1 - Label	ROI2 - Network	ROI2 - Label
Memory retrieval	L Precuneus & L BA 7	Default mode	L Middle Temporal Gyrus
Cingulo opercular Task Control	R Superior Temporal Gyrus	Default mode	L Middle Temporal Gyrus
Salience	R Superior Frontal Gyrus	Visual	L Fusiform Gyrus & L BA 19
Salience	L Cingulate Gyrus	Visual	R Cuneus
Sensory somatomotor Hand	L Postcentral Gyrus	Subcortical	R Thalamus
Visual	R Cuneus & R BA 19	Visual	R Lingual Gyrus
Cingulo opercular Task Control	L Superior Frontal Gyrus	Fronto parietal Task Control	R Superior Frontal Gyrus & R BA 11
Subcortical	R Lentiform Nucleus	Sensory somatomotor Hand	L Cingulate Gyrus
Cingulo opercular Task Control	L Claustrum	Sensory somatomotor Hand	R Cingulate Gyrus & R BA 24
Subcortical	L Lentiform Nucleus & L Putamen	Default mode	L Middle Temporal Gyrus
Default mode	L Posterior Cingulate	Visual	R Middle Occipital Gyrus
Sensory somatomotor Hand	R Paracentral Lobule	Subcortical	L Lentiform Nucleus & L Putamen
Sensory somatomotor Hand	R Postcentral Gyrus & R BA 5	Fronto parietal Task Control	R Inferior Parietal Lobule
Fronto parietal Task Control	R Inferior Parietal Lobule	Default mode	R Anterior Cingulate
Auditory	R Insula & R BA 13	Subcortical	R Thalamus
Salience	R Superior Frontal Gyrus & R BA 10	Salience	R Middle Frontal Gyrus

Visual	R Middle Occipital Gyrus	Visual	L Middle Occipital Gyrus
Sensory somatomotor Hand	L Postcentral Gyrus & L BA 5	Default mode	L Inferior Frontal Gyrus & L BA 47
Sensory somatomotor Hand	L Precentral Gyrus	Fronto parietal Task Control	R Superior Frontal Gyrus & R BA 11
Cingulo opercular Task Control	R Superior Frontal Gyrus & R BA 6	Subcortical	R Lentiform Nucleus
Default mode	L Precuneus & L BA 7	Sensory somatomotor Mouth	L Precentral Gyrus
Salience	R Middle Frontal Gyrus	Fronto parietal Task Control	R Middle Frontal Gyrus
Visual	R Lingual Gyrus	Default mode	R Pyramis
Default mode	L Middle Temporal Gyrus	Visual	R Superior Occipital Gyrus
Auditory	R Postcentral Gyrus	Default mode	R Supramarginal Gyrus
Default mode	R Medial Frontal Gyrus	Default mode	L Middle Temporal Gyrus & L BA 21
Auditory	L Inferior Parietal Lobule	Sensory somatomotor Mouth	L Postcentral Gyrus
Default mode	L Superior Frontal Gyrus & L BA 8	Salience	R Superior Frontal Gyrus
Sensory somatomotor Hand	L Postcentral Gyrus & L BA 5	Ventral attention	L Inferior Frontal Gyrus & L BA 47
Cerebellar	R Culmen	Default mode	L Superior Frontal Gyrus
Cingulo opercular Task Control	L Claustrum	Salience	R Cingulate Gyrus
Visual	R Middle Occipital Gyrus & R BA 19	Visual	R Middle Occipital Gyrus
Cingulo opercular Task Control	R Superior Temporal Gyrus	Ventral attention	L Superior Temporal Gyrus & L BA 22
Default mode	L Cingulate Gyrus & L BA 31	Subcortical	L Thalamus
Sensory somatomotor Mouth	R Precentral Gyrus	Salience	L Superior Frontal Gyrus & L BA 10
Default mode	R Medial Frontal Gyrus	Default mode	L Superior Frontal Gyrus
Dorsal attention	L Inferior Parietal Lobule	Visual	R Fusiform Gyrus
Default mode	L Superior Frontal Gyrus & L BA 8	Default mode	R Anterior Cingulate

Subcortical	R Lentiform Nucleus	Visual	L Middle Occipital Gyrus
Visual	L Inferior Occipital Gyrus & L BA 18	Default mode	R Anterior Cingulate
Salience	R Superior Frontal Gyrus	Salience	R Inferior Frontal Gyrus & R BA 45
Default mode	L Superior Occipital Gyrus	Cerebellar	R Culmen
Sensory somatomotor Mouth	L Postcentral Gyrus	Subcortical	L Thalamus
Salience	R Superior Frontal Gyrus	Default mode	L Superior Frontal Gyrus
Dorsal attention	L Fusiform Gyrus	Fronto parietal Task Control	R Inferior Temporal Gyrus & R BA 20
Default mode	L Middle Temporal Gyrus	Default mode	L Superior Frontal Gyrus & L BA 10
Cerebellar	L Declive	Fronto parietal Task Control	L Medial Frontal Gyrus & L BA 8
Sensory somatomotor Hand	Not Found	Default mode	L Medial Frontal Gyrus & L BA 6
Subcortical	R Thalamus & R Ventral Anterior Nucleus	Visual	L Cuneus & L BA 18
Default mode	R Middle Temporal Gyrus & R BA 39	Sensory somatomotor Hand	R Postcentral Gyrus & R BA 5
Dorsal attention	L Inferior Parietal Lobule	Default mode	R Pyramis
Auditory	L Inferior Parietal Lobule	Visual	L Middle Occipital Gyrus & L BA 19
Default mode	R Precuneus & R BA 31	Auditory	L Inferior Parietal Lobule
Cingulo opercular Task Control	R Postcentral Gyrus & R BA 2	Subcortical	L Lentiform Nucleus & L Putamen
Auditory	L Superior Temporal Gyrus & L BA 42	Cingulo opercular Task Control	L Precentral Gyrus & L BA 44
Default mode	L Precuneus & L BA 7	Default mode	L Middle Temporal Gyrus & L BA 21
Auditory	R Insula	Memory retrieval	L Precuneus & L BA 7
Dorsal attention	R Middle Temporal Gyrus	Fronto parietal Task Control	R Inferior Parietal Lobule
Cingulo opercular Task Control	L Precentral Gyrus & L BA 44	Subcortical	R Thalamus

Ventral attention	R Middle Temporal Gyrus	Default mode	R Superior Frontal Gyrus
Auditory	L Inferior Parietal Lobule	Visual	R Middle Occipital Gyrus
Cerebellar	L Declive	Salience	R Inferior Frontal Gyrus & R BA 45
Fronto parietal Task Control	R Superior Frontal Gyrus & R BA 11	Visual	L Middle Occipital Gyrus
Cingulo opercular Task Control	L Superior Temporal Gyrus & L BA 22	Cingulo opercular Task Control	L Superior Frontal Gyrus
Fronto parietal Task Control	R Superior Frontal Gyrus & R BA 11	Visual	R Fusiform Gyrus
Visual	R Middle Occipital Gyrus	Visual	R Cuneus & R BA 19
Cingulo opercular Task Control	L Claustrum	Subcortical	L Lentiform Nucleus & L Putamen
Default mode	L Middle Temporal Gyrus & L BA 21	Default mode	R Superior Frontal Gyrus
Sensory somatomotor Hand	L Postcentral Gyrus	Default mode	L Inferior Frontal Gyrus & L BA 47
Salience	R Superior Frontal Gyrus	Default mode	L Medial Frontal Gyrus
Sensory somatomotor Mouth	L Precentral Gyrus	Fronto parietal Task Control	R Middle Frontal Gyrus & R BA 6
Salience	R Superior Frontal Gyrus	Default mode	L Medial Frontal Gyrus & L BA 9
Visual	L Inferior Occipital Gyrus & L BA 18	Visual	R Cuneus & R BA 19
Default mode	R Superior Temporal Gyrus	Default mode	L Middle Temporal Gyrus
Default mode	L Middle Temporal Gyrus	Subcortical	R Lentiform Nucleus & R Putamen
Default mode	R Precuneus & R BA 31	Cerebellar	L Declive
Default mode	R Superior Temporal Gyrus	Fronto parietal Task Control	R Middle Frontal Gyrus & R BA 11
Dorsal attention	L Fusiform Gyrus	Default mode	R Middle Temporal Gyrus
Subcortical	R Thalamus & R Ventral Anterior Nucleus	Visual	R Cuneus & R BA 23
Fronto parietal Task Control	R Superior Frontal Gyrus & R BA 11	Visual	L Inferior Occipital Gyrus & L BA 18

Cingulo opercular Task Control	R Postcentral Gyrus & R BA 2	Subcortical	R Lentiform Nucleus
Salience	R Superior Frontal Gyrus	Salience	R Inferior Frontal Gyrus & R BA 47
Default mode	R Middle Temporal Gyrus & R BA 39	Visual	R Cuneus & R BA 19
Auditory	L Insula	Subcortical	R Lentiform Nucleus & R Putamen
Salience	R Insula & R BA 13	Default mode	R Superior Frontal Gyrus
Visual	L Middle Occipital Gyrus	Visual	R Fusiform Gyrus
Salience	R Middle Frontal Gyrus	Default mode	R Middle Temporal Gyrus
Fronto parietal Task Control	R Superior Frontal Gyrus & R BA 11	Visual	R Middle Occipital Gyrus
Ventral attention	R Middle Temporal Gyrus	Visual	R Declive
Fronto parietal Task Control	R Superior Frontal Gyrus & R BA 11	Visual	L Middle Occipital Gyrus
Default mode	R Superior Temporal Gyrus	Ventral attention	R Middle Temporal Gyrus
Subcortical	R Thalamus	Fronto parietal Task Control	R Middle Frontal Gyrus
Cerebellar	L Declive	Subcortical	R Lentiform Nucleus
Sensory somatomotor Mouth	R Precentral Gyrus	Fronto parietal Task Control	R Superior Frontal Gyrus & R BA 11
Ventral attention	R Superior Temporal Gyrus & R BA 22	Memory retrieval	L Precuneus & L BA 7
Subcortical	R Lentiform Nucleus	Default mode	R Superior Frontal Gyrus
Dorsal attention	L Fusiform Gyrus	Visual	L Middle Occipital Gyrus
Default mode	R Middle Temporal Gyrus & R BA 39	Visual	L Middle Occipital Gyrus & L BA 19
Sensory somatomotor Hand	L Precentral Gyrus	Fronto parietal Task Control	R Superior Frontal Gyrus & R BA 11
Default mode	L Superior Occipital Gyrus	Default mode	L Parahippocampal Gyrus & L BA 36
Fronto parietal Task Control	L Superior Frontal Gyrus	Visual	R Fusiform Gyrus
Default mode	R Middle Temporal	Sensory	R Postcentral Gyrus

	Gyrus & R BA 39	somatomotor Hand	& R BA 3
Cingulo opercular Task Control	L Precentral Gyrus & L BA 44	Sensory somatomotor Hand	L Precentral Gyrus
Default mode	R Medial Frontal Gyrus	Default mode	L Superior Frontal Gyrus
Default mode	R Middle Temporal Gyrus	Default mode	L Medial Frontal Gyrus & L BA 6
Auditory	R Insula	Default mode	L Middle Temporal Gyrus
Salience	R Superior Frontal Gyrus	Default mode	R Superior Frontal Gyrus
Salience	R Inferior Frontal Gyrus & R BA 45	Fronto parietal Task Control	R Middle Frontal Gyrus
Visual	L Cuneus & L BA 18	Visual	L Middle Occipital Gyrus
Auditory	R Postcentral Gyrus	Salience	R Supramarginal Gyrus
Salience	R Superior Frontal Gyrus	Salience	R Insula & R BA 13
Subcortical	L Lentiform Nucleus & L Putamen	Fronto parietal Task Control	R Superior Frontal Gyrus & R BA 11
Default mode	L Precuneus	Default mode	L Medial Frontal Gyrus & L BA 6
Subcortical	R Lentiform Nucleus	Sensory somatomotor Hand	L Postcentral Gyrus
Cingulo opercular Task Control	R Insula	Dorsal attention	Superior Parietal Lobule
Default mode	L Middle Temporal Gyrus & L BA 21	Default mode	R Superior Frontal Gyrus
Default mode	R Angular Gyrus	Default mode	R Superior Frontal Gyrus
Salience	L Superior Frontal Gyrus & L BA 10	Default mode	R Middle Temporal Gyrus & R BA 21
Default mode	R Medial Frontal Gyrus	Visual	R Middle Occipital Gyrus
Sensory somatomotor Mouth	L Precentral Gyrus	Subcortical	R Thalamus
Sensory somatomotor Hand	L Paracentral Lobule	Default mode	L Superior Frontal Gyrus & L BA 10
Default mode	L Superior Frontal Gyrus	Cingulo opercular Task Control	R Insula
Cingulo opercular Task Control	L Precentral Gyrus & L BA 44	Sensory somatomotor Hand	L Postcentral Gyrus
Default mode	R Middle Temporal Gyrus & R BA 39	Sensory somatomotor Hand	R Medial Frontal Gyrus

Subcortical	R Thalamus	Default mode	R Inferior Temporal Gyrus
Subcortical	R Lentiform Nucleus	Sensory somatomotor Hand	L Postcentral Gyrus
Subcortical	L Thalamus	Fronto parietal Task Control	L Middle Frontal Gyrus & L BA 10
Sensory somatomotor Mouth	R Precentral Gyrus	Fronto parietal Task Control	L Middle Frontal Gyrus & L BA 46
Visual	R Declive	Default mode	L Fusiform Gyrus & L BA 20
Cingulo opercular Task Control	L Claustrum	Visual	R Precuneus & R BA 31
Cingulo opercular Task Control	R Superior Temporal Gyrus	Sensory somatomotor Hand	R Postcentral Gyrus
Auditory	R Insula	Memory retrieval	L Cingulate Gyrus & L BA 31
Cingulo opercular Task Control	L Precentral Gyrus & L BA 44	Sensory somatomotor Hand	R Postcentral Gyrus
Dorsal attention	L Fusiform Gyrus	Default mode	R Anterior Cingulate
Default mode	R Medial Frontal Gyrus	Default mode	R Superior Frontal Gyrus
Default mode	L Superior Frontal Gyrus	Default mode	R Inferior Temporal Gyrus
Default mode	L Middle Temporal Gyrus	Default mode	R Inferior Temporal Gyrus
Visual	L Lingual Gyrus	Default mode	R Superior Frontal Gyrus
Visual	L Inferior Occipital Gyrus & L BA 18	Default mode	L Parahippocampal Gyrus & L BA 36
Dorsal attention	L Middle Temporal Gyrus	Subcortical	R Lentiform Nucleus
Default mode	L Cingulate Gyrus & L BA 31	Visual	R Middle Occipital Gyrus & R BA 19
Sensory somatomotor Hand	L Postcentral Gyrus & L BA 5	Default mode	L Medial Frontal Gyrus & L BA 6
Sensory somatomotor Mouth	L Precentral Gyrus	Fronto parietal Task Control	R Superior Frontal Gyrus & R BA 11
Sensory somatomotor Hand	L Postcentral Gyrus & L BA 5	Default mode	R Inferior Temporal Gyrus
Salience	R Middle Frontal Gyrus	Default mode	L Medial Frontal Gyrus
Subcortical	R Lentiform Nucleus	Memory retrieval	L Cingulate Gyrus & L BA 31

Fronto parietal Task Control	L Inferior Frontal Gyrus	Default mode	R Inferior Temporal Gyrus
Fronto parietal Task Control	R Middle Frontal Gyrus	Sensory somatomotor Hand	L Precuneus
Fronto parietal Task Control	R Superior Frontal Gyrus & R BA 11	Visual	L Middle Occipital Gyrus & L BA 19
Visual	L Middle Occipital Gyrus	Default mode	L Parahippocampal Gyrus & L BA 36
Sensory somatomotor Mouth	L Precentral Gyrus	Subcortical	R Lentiform Nucleus
Sensory somatomotor Mouth	L Postcentral Gyrus	Dorsal attention	L Precuneus & L BA 19
Fronto parietal Task Control	L Medial Frontal Gyrus & L BA 8	Default mode	R Middle Temporal Gyrus
Auditory	L Precentral Gyrus & L BA 43	Subcortical	R Lentiform Nucleus
Fronto parietal Task Control	R Superior Frontal Gyrus & R BA 11	Visual	L Middle Occipital Gyrus & L BA 19
Cingulo opercular Task Control	L Superior Frontal Gyrus	Sensory somatomotor Mouth	L Precentral Gyrus
Subcortical	L Lentiform Nucleus & L Putamen	Fronto parietal Task Control	L Inferior Parietal Lobule & L BA 40
Auditory	L Superior Temporal Gyrus	Fronto parietal Task Control	L Medial Frontal Gyrus & L BA 8
Default mode	R Middle Temporal Gyrus & R BA 39	Sensory somatomotor Hand	L Postcentral Gyrus
Sensory somatomotor Hand	L Postcentral Gyrus & L BA 5	Default mode	R Inferior Frontal Gyrus
Salience	R Superior Frontal Gyrus & R BA 10	Fronto parietal Task Control	R Middle Frontal Gyrus & R BA 11
Subcortical	R Thalamus	Sensory somatomotor Hand	R Precentral Gyrus
Sensory somatomotor Mouth	L Precentral Gyrus	Sensory somatomotor Hand	L Postcentral Gyrus
Ventral attention	R Inferior Parietal Lobule & R BA 40	Memory retrieval	L Precuneus & L BA 7
Ventral attention	R Superior Temporal Gyrus & R BA 22	Sensory somatomotor Hand	Not Found
Visual	R Fusiform Gyrus	Default mode	L Fusiform Gyrus & L BA 20
Sensory	R Postcentral Gyrus	Dorsal attention	L Inferior Parietal

somatomotor Hand	& R BA 3		Lobule
Visual	L Fusiform Gyrus & L BA 19	Ventral attention	L Superior Frontal Gyrus & L BA 6
Sensory somatomotor Mouth	L Precentral Gyrus	Dorsal attention	L Middle Frontal Gyrus
Visual	R Lingual Gyrus	Visual	R Fusiform Gyrus
Default mode	R Superior Frontal Gyrus & R BA 8	Default mode	L Medial Frontal Gyrus & L BA 6
Sensory somatomotor Hand	L Paracentral Lobule	Default mode	R Inferior Temporal Gyrus
Sensory somatomotor Hand	L Postcentral Gyrus & L BA 5	Ventral attention	R Inferior Frontal Gyrus
Salience	R Supramarginal Gyrus	Sensory somatomotor Hand	R Cingulate Gyrus & R BA 24
Default mode	R Middle Temporal Gyrus & R BA 39	Sensory somatomotor Hand	R Paracentral Lobule
Dorsal attention	L Fusiform Gyrus	Default mode	R Inferior Temporal Gyrus
Sensory somatomotor Hand	L Postcentral Gyrus & L BA 5	Fronto parietal Task Control	L Inferior Frontal Gyrus
Salience	R Inferior Frontal Gyrus & R BA 47	Default mode	L Medial Frontal Gyrus & L BA 6
Cingulo opercular Task Control	R Postcentral Gyrus & R BA 2	Cingulo opercular Task Control	L Medial Frontal Gyrus & R BA 6
Dorsal attention	Superior Parietal Lobule	Fronto parietal Task Control	R Inferior Parietal Lobule & R BA 40
Default mode	L Angular Gyrus & L BA 39	Visual	R Cuneus
Sensory somatomotor Mouth	R Precentral Gyrus & R BA 6	Subcortical	R Lentiform Nucleus
Salience	R Inferior Frontal Gyrus & R BA 45	Default mode	L Medial Frontal Gyrus & L BA 9
Dorsal attention	L Fusiform Gyrus	Visual	R Fusiform Gyrus
Cingulo opercular Task Control	R Superior Frontal Gyrus & R BA 6	Salience	R Middle Frontal Gyrus
Subcortical	L Thalamus	Visual	R Cuneus & R BA 19
Sensory somatomotor Hand	R Precentral Gyrus	Fronto parietal Task Control	R Superior Frontal Gyrus & R BA 11
Cingulo opercular Task Control	R Insula	Default mode	R Superior Frontal Gyrus
Cingulo opercular Task Control	L Claustrum	Visual	R Superior Occipital Gyrus
Sensory	R Postcentral Gyrus	Sensory	L Postcentral

somatomotor Hand	& R BA 3	somatomotor Hand	Gyrus & L BA 5
Default mode	R Superior Temporal Gyrus	Memory retrieval	L Cingulate Gyrus & L BA 31
Fronto parietal Task Control	R Inferior Temporal Gyrus & R BA 20	Default mode	L Superior Frontal Gyrus
Dorsal attention	L Fusiform Gyrus	Visual	R Middle Occipital Gyrus & R BA 19
Sensory somatomotor Mouth	L Precentral Gyrus	Sensory somatomotor Hand	L Postcentral Gyrus & L BA 5
Visual	L Fusiform Gyrus & L BA 19	Visual	R Fusiform Gyrus
Salience	R Middle Frontal Gyrus	Fronto parietal Task Control	R Middle Frontal Gyrus
Default mode	R Superior Temporal Gyrus	Salience	R Inferior Frontal Gyrus & R BA 47
Sensory somatomotor Mouth	L Postcentral Gyrus	Visual	L Middle Occipital Gyrus & L BA 19
Sensory somatomotor Hand	L Cingulate Gyrus	Default mode	L Middle Temporal Gyrus
Default mode	R Middle Temporal Gyrus & R BA 39	Salience	R Inferior Frontal Gyrus & R BA 45
Auditory	R Postcentral Gyrus	Fronto parietal Task Control	R Superior Frontal Gyrus & R BA 11
Sensory somatomotor Mouth	R Precentral Gyrus	Dorsal attention	R Precuneus & R BA 7
Salience	R Middle Frontal Gyrus	Ventral attention	L Superior Frontal Gyrus & L BA 6
Sensory somatomotor Mouth	L Postcentral Gyrus	Visual	L Middle Occipital Gyrus
Sensory somatomotor Mouth	R Precentral Gyrus & R BA 6	Sensory somatomotor Hand	R Postcentral Gyrus
Default mode	L Posterior Cingulate & L BA 29	Cingulo opercular Task Control	R Medial Frontal Gyrus
Cingulo opercular Task Control	R Insula	Dorsal attention	L Fusiform Gyrus
Default mode	L Superior Temporal Gyrus & L BA 39	Salience	L Anterior Cingulate
Sensory somatomotor Hand	L Postcentral Gyrus & L BA 5	Salience	R Middle Frontal Gyrus & R BA 6
Default mode	R Middle Temporal	Cingulo opercular	L Superior Frontal

	Gyrus & R BA 39	Task Control	Gyrus
Cingulo opercular Task Control	L Superior Frontal Gyrus	Subcortical	R Lentiform Nucleus
Sensory somatomotor Mouth	L Precentral Gyrus	Fronto parietal Task Control	L Superior Parietal Lobule & L BA 7
Salience	R Insula & R BA 13	Salience	R Supramarginal Gyrus
Auditory	L Inferior Parietal Lobule	Salience	L Superior Frontal Gyrus & L BA 10
Subcortical	L Lentiform Nucleus & L Putamen	Fronto parietal Task Control	R Middle Frontal Gyrus
Default mode	R Superior Frontal Gyrus & R BA 8	Subcortical	R Lentiform Nucleus
Auditory	R Superior Temporal Gyrus & R BA 42	Sensory somatomotor Hand	R Postcentral Gyrus & R BA 3
Dorsal attention	L Fusiform Gyrus	Visual	R Middle Occipital Gyrus
Fronto parietal Task Control	R Superior Frontal Gyrus & R BA 11	Default mode	L Middle Temporal Gyrus
Sensory somatomotor Mouth	L Postcentral Gyrus	Fronto parietal Task Control	R Middle Frontal Gyrus & R BA 6
Sensory somatomotor Hand	R Postcentral Gyrus & R BA 3	Salience	R Inferior Frontal Gyrus & R BA 47
Dorsal attention	L Fusiform Gyrus	Visual	R Middle Occipital Gyrus
Cingulo opercular Task Control	L Medial Frontal Gyrus & R BA 6	Salience	R Supramarginal Gyrus
Sensory somatomotor Hand	L Postcentral Gyrus & L BA 5	Sensory somatomotor Hand	R Postcentral Gyrus & R BA 5
Visual	R Cuneus & R BA 19	Default mode	R Superior Frontal Gyrus
Dorsal attention	L Inferior Parietal Lobule	Sensory somatomotor Hand	R Postcentral Gyrus & R BA 5
Dorsal attention	L Fusiform Gyrus	Visual	R Superior Occipital Gyrus
Sensory somatomotor Hand	R Postcentral Gyrus	Fronto parietal Task Control	L Superior Parietal Lobule & L BA 7
Dorsal attention	L Middle Frontal Gyrus	Ventral attention	R Inferior Frontal Gyrus
Ventral attention	R Superior Temporal Gyrus & R BA 22	Sensory somatomotor Hand	R Postcentral Gyrus

Dorsal attention	L Middle Frontal Gyrus	Sensory somatomotor Hand	L Medial Frontal Gyrus
Sensory somatomotor Hand	L Precuneus	Default mode	R Middle Temporal Gyrus
Sensory somatomotor Mouth	L Precentral Gyrus	Fronto parietal Task Control	L Medial Frontal Gyrus & L BA 8
Dorsal attention	L Fusiform Gyrus	Dorsal attention	R Middle Temporal Gyrus
Auditory	R Superior Temporal Gyrus & R BA 42	Memory retrieval	R Precuneus & R BA 7
Ventral attention	R Inferior Parietal Lobule & R BA 40	Sensory somatomotor Hand	Not Found
Sensory somatomotor Mouth	L Precentral Gyrus	Visual	L Middle Occipital Gyrus
Cerebellar	R Declive	Default mode	L Superior Frontal Gyrus

Supplementary Table 3. The list of all ROIs arranged in order of importance in contribution towards classification between pre- and post-therapy stages specified by the network they belong to and the anatomical label as per MNI coordinates.

Rank	ROI Network	ROI Label
1	Fronto parietal Task Control	L Middle Frontal Gyrus
2	Fronto parietal Task Control	R Superior Frontal Gyrus & R BA 11
3	Sensory somatomotor Hand	R Precentral Gyrus
4	Subcortical	R Lentiform Nucleus
5	Default mode	L Superior Frontal Gyrus
6	Visual	R Cuneus & R BA 19
7	Default mode	R Medial Frontal Gyrus & R BA 10
8	Sensory somatomotor Mouth	L Precentral Gyrus
9	Default mode	L Middle Temporal Gyrus
10	Fronto parietal Task Control	R Middle Frontal Gyrus
11	Subcortical	L Thalamus
12	Subcortical	R Thalamus
13	Visual	R Lingual Gyrus
14	Default mode	L Superior Frontal Gyrus & L BA 6
15	Default mode	L Parahippocampal Gyrus & L BA 36
16	Dorsal attention	L Fusiform Gyrus
17	Default mode	L Middle Temporal Gyrus
18	Default mode	R Middle Temporal Gyrus & R BA 39
19	Salience	R Superior Frontal Gyrus
20	Default mode	L Anterior Cingulate
21	Fronto parietal Task Control	R Inferior Temporal Gyrus & R BA 20

22	Sensory somatomotor Hand	L Postcentral Gyrus & L BA 5
23	Visual	R Declive
24	Sensory somatomotor Hand	R Postcentral Gyrus & R BA 5
25	Default mode	L Medial Frontal Gyrus & L BA 6
26	Default mode	R Precuneus & R BA 31
27	Visual	R Cuneus & R BA 19
28	Visual	R Lingual Gyrus
29	Visual	R Middle Occipital Gyrus
30	Subcortical	L Lentiform Nucleus & L Putamen
31	Default mode	R Superior Frontal Gyrus
32	Default mode	L Precuneus
33	Ventral attention	R Inferior Parietal Lobule & R BA 40
34	Default mode	L Inferior Frontal Gyrus & L BA 47
35	Visual	L Fusiform Gyrus & L BA 19
36	Visual	R Cuneus
37	Default mode	R Posterior Cingulate
38	Default mode	R Pyramis
39	Visual	R Middle Occipital Gyrus
40	Cingulo opercular Task Control	L Cingulate Gyrus
41	Default mode	R Superior Frontal Gyrus
42	Default mode	R Inferior Temporal Gyrus
43	Fronto parietal Task Control	R Middle Frontal Gyrus & R BA 6
44	Visual	L Lingual Gyrus
45	Salience	L Superior Frontal Gyrus & L BA 10
46	Visual	R Cuneus & R BA 17
47	Subcortical	R Lentiform Nucleus & R Putamen
48	Sensory somatomotor Hand	L Postcentral Gyrus
49	Auditory	L Insula
50	Default mode	R Anterior Cingulate & R BA 32
51	Default mode	L Superior Occipital Gyrus
52	Default mode	R Medial Frontal Gyrus & R BA 9
53	Default mode	L Parahippocampal Gyrus & L BA 30
54	Visual	L Middle Occipital Gyrus
55	Visual	R Cuneus & R BA 23
56	Fronto parietal Task Control	R Inferior Parietal Lobule & R BA 40
57	Visual	R Culmen
58	Auditory	R Insula & R BA 13
59	Cingulo opercular Task Control	L Claustrum
60	Subcortical	R Thalamus
61	Visual	R Middle Occipital Gyrus
62	Visual	L Middle Occipital Gyrus & L BA 19

63	Auditory	R Precentral Gyrus & R BA 43
64	Default mode	L Superior Frontal Gyrus & L BA 8
65	Fronto parietal Task Control	L Inferior Frontal Gyrus
66	Sensory somatomotor Hand	L Precentral Gyrus
67	Visual	L Inferior Occipital Gyrus & L BA 18
68	Default mode	L Angular Gyrus & L BA 39
69	Cingulo opercular Task Control	L Superior Frontal Gyrus
70	Sensory somatomotor Mouth	L Postcentral Gyrus
71	Sensory somatomotor Hand	R Postcentral Gyrus
72	Sensory somatomotor Hand	Not Found
73	Salience	R Superior Frontal Gyrus & R BA 10
74	Auditory	L Inferior Parietal Lobule
75	Salience	R Middle Frontal Gyrus
76	Memory retrieval	L Precuneus & L BA 7
77	Dorsal attention	R Middle Frontal Gyrus
78	Auditory	R Superior Temporal Gyrus & R BA 42
79	Subcortical	R Thalamus & R Ventral Anterior Nucleus
80	Cingulo opercular Task Control	L Precentral Gyrus & L BA 44
81	Fronto parietal Task Control	R Inferior Parietal Lobule
82	Visual	R Middle Occipital Gyrus & R BA 19
83	Salience	L Insula & L BA 13
84	Visual	L Lingual Gyrus & L BA 19
85	Cingulo opercular Task Control	R Superior Frontal Gyrus
86	Default mode	R Middle Temporal Gyrus
87	Sensory somatomotor Mouth	R Precentral Gyrus
88	Dorsal attention	L Inferior Parietal Lobule
89	Fronto parietal Task Control	L Middle Frontal Gyrus & L BA 10
90	Auditory	R Postcentral Gyrus
91	Fronto parietal Task Control	R Middle Frontal Gyrus
92	Visual	L Cuneus & L BA 18
93	Ventral attention	L Superior Temporal Gyrus & L BA 22
94	Fronto parietal Task Control	L Inferior Parietal Lobule
95	Fronto parietal Task Control	R Middle Frontal Gyrus
96	Dorsal attention	Superior Parietal Lobule
97	Visual	L Middle Occipital Gyrus & L BA 19
98	Sensory somatomotor Hand	L Precentral Gyrus & L BA 4
99	Cingulo opercular Task Control	R Medial Frontal Gyrus
100	Sensory somatomotor Hand	Not Found
101	Sensory somatomotor Hand	L Precentral Gyrus
102	Visual	R Fusiform Gyrus
103	Fronto parietal Task Control	L Middle Frontal Gyrus & L BA 6

104	Visual	L Lingual Gyrus
105	Salience	R Supramarginal Gyrus
106	Cingulo opercular Task Control	L Medial Frontal Gyrus & R BA 6
107	Cerebellar	L Declive
108	Cerebellar	L Culmen
109	Visual	R Fusiform Gyrus
110	Default mode	L Middle Temporal Gyrus
111	Default mode	L Superior Frontal Gyrus
112	Dorsal attention	L Precuneus & L BA 19
113	Default mode	R Supramarginal Gyrus
114	Default mode	L Superior Frontal Gyrus
115	Default mode	L Medial Frontal Gyrus
116	Fronto parietal Task Control	R Inferior Parietal Lobule
117	Auditory	L Precentral Gyrus & L BA 43
118	Salience	R Inferior Frontal Gyrus & R BA 45
119	Sensory somatomotor Hand	L Postcentral Gyrus
120	Default mode	R Superior Temporal Gyrus
121	Visual	L Cuneus
122	Cingulo opercular Task Control	R Insula
123	Subcortical	R Lentiform Nucleus & R Putamen
124	Fronto parietal Task Control	R Middle Frontal Gyrus
125	Default mode	R Parahippocampal Gyrus
126	Sensory somatomotor Hand	R Postcentral Gyrus & R BA 3
127	Sensory somatomotor Hand	L Postcentral Gyrus
128	Subcortical	L Lentiform Nucleus & L Putamen
129	Memory retrieval	L Cingulate Gyrus & L BA 31
130	Visual	L Middle Occipital Gyrus
131	Visual	R Superior Occipital Gyrus
132	Cingulo opercular Task Control	R Insula
133	Default mode	R Precuneus & R BA 31
134	Fronto parietal Task Control	L Superior Parietal Lobule & L BA 7
135	Default mode	L Middle Temporal Gyrus
136	Visual	L Cuneus
137	Sensory somatomotor Hand	L Medial Frontal Gyrus
138	Default mode	L Fusiform Gyrus & L BA 20
139	Sensory somatomotor Hand	R Paracentral Lobule
140	Subcortical	L Thalamus & L Medial Dorsal Nucleus
141	Sensory somatomotor Hand	R Postcentral Gyrus & R BA 3
142	Memory retrieval	R Precuneus & R BA 7
143	Default mode	R Anterior Cingulate
144	Cingulo opercular Task	R Superior Temporal Gyrus

	Control	
145	Default mode	L Middle Temporal Gyrus & L BA 21
146	Auditory	L Superior Temporal Gyrus & L BA 42
147	Default mode	L Middle Temporal Gyrus
148	Dorsal attention	R Precuneus & R BA 7
149	Subcortical	L Thalamus
150	Default mode	R Medial Frontal Gyrus
151	Default mode	L Posterior Cingulate
152	Cingulo opercular Task Control	R Postcentral Gyrus & R BA 2
153	Salience	R Insula & R BA 13
154	Default mode	L Cingulate Gyrus & L BA 31
155	Memory retrieval	R Precuneus
156	Default mode	L Anterior Cingulate & L BA 10
157	Ventral attention	L Superior Frontal Gyrus & L BA 6
158	Salience	R Middle Frontal Gyrus
159	Default mode	R Superior Frontal Gyrus
160	Salience	L Middle Frontal Gyrus & L BA 10
161	Sensory somatomotor Hand	L Cingulate Gyrus
162	Default mode	R Middle Temporal Gyrus
163	Dorsal attention	L Middle Temporal Gyrus
164	Fronto parietal Task Control	L Medial Frontal Gyrus & L BA 8
165	Salience	R Inferior Frontal Gyrus & R BA 47
166	Sensory somatomotor Hand	L Paracentral Lobule
167	Sensory somatomotor Hand	R Cingulate Gyrus & R BA 24
168	Cerebellar	R Declive
169	Dorsal attention	R Superior Parietal Lobule & R BA 7
170	Sensory somatomotor Mouth	R Precentral Gyrus & R BA 6
171	Default mode	R Inferior Frontal Gyrus
172	Default mode	L Middle Temporal Gyrus
173	Visual	L Middle Occipital Gyrus
174	Ventral attention	R Middle Temporal Gyrus
175	Fronto parietal Task Control	R Middle Frontal Gyrus & R BA 11
176	Auditory	R Insula
177	Fronto parietal Task Control	L Inferior Parietal Lobule & L BA 40
178	Auditory	L Superior Temporal Gyrus
179	Cingulo opercular Task Control	R Superior Frontal Gyrus & R BA 6
180	Salience	R Cingulate Gyrus
181	Default mode	L Medial Frontal Gyrus & L BA 10
182	Ventral attention	L Inferior Frontal Gyrus & L BA 47
183	Subcortical	L Lentiform Nucleus & L Putamen
184	Fronto parietal Task Control	R Middle Frontal Gyrus
185	Default mode	R Medial Frontal Gyrus

186	Default mode	L Superior Temporal Gyrus & L BA 39
187	Fronto parietal Task Control	R Inferior Parietal Lobule
188	Default mode	L Medial Frontal Gyrus & L BA 9
189	Default mode	R Middle Temporal Gyrus & R BA 21
190	Fronto parietal Task Control	L Inferior Frontal Gyrus & L BA 9
191	Default mode	L Superior Frontal Gyrus
192	Sensory somatomotor Hand	R Superior Frontal Gyrus
193	Sensory somatomotor Hand	L Inferior Parietal Lobule & L BA 40
194	Dorsal attention	R Precuneus & R BA 7
195	Ventral attention	R Superior Temporal Gyrus & R BA 22
196	Cingulo opercular Task Control	L Superior Temporal Gyrus & L BA 22
197	Default mode	R Superior Frontal Gyrus & R BA 8
198	Salience	R Middle Frontal Gyrus & R BA 6
199	Sensory somatomotor Hand	R Precentral Gyrus
200	Sensory somatomotor Hand	L Precuneus
201	Salience	L Anterior Cingulate
202	Sensory somatomotor Hand	R Postcentral Gyrus
203	Dorsal attention	L Middle Frontal Gyrus
204	Sensory somatomotor Hand	R Postcentral Gyrus
205	Sensory somatomotor Hand	R Postcentral Gyrus
206	Cerebellar	R Culmen
207	Default mode	L Precuneus & L BA 7
208	Salience	L Cingulate Gyrus
209	Default mode	L Superior Frontal Gyrus & L BA 10
210	Memory retrieval	R Cingulate Gyrus
211	Auditory	L Postcentral Gyrus
212	Salience	R Paracentral Lobule
213	Sensory somatomotor Hand	L Paracentral Lobule
214	Fronto parietal Task Control	L Middle Frontal Gyrus & L BA 46
215	Sensory somatomotor Hand	L Postcentral Gyrus
216	Default mode	R Angular Gyrus
217	Subcortical	R Lentiform Nucleus & R Putamen
218	Visual	R Precuneus & R BA 31
219	Visual	L Precuneus & L BA 7
220	Sensory somatomotor Hand	R Precentral Gyrus & R BA 4
221	Dorsal attention	R Middle Temporal Gyrus
222	Ventral attention	L Superior Temporal Gyrus
223	Auditory	R Superior Temporal Gyrus
224	Ventral attention	R Inferior Frontal Gyrus
225	Salience	R Cingulate Gyrus & R BA 32
226	Fronto parietal Task Control	L Superior Frontal Gyrus
227	Sensory somatomotor Hand	R Medial Frontal Gyrus

228	Auditory	L Superior Temporal Gyrus & L BA 41
229	Default mode	L Posterior Cingulate & L BA 29
230	Cingulo opercular Task Control	L Cingulate Gyrus
231	Salience	L Medial Frontal Gyrus & L BA 6
232	Ventral attention	R Superior Temporal Gyrus
233	Default mode	R Superior Frontal Gyrus & R BA 8
234	Fronto parietal Task Control	L Middle Frontal Gyrus & L BA 9
235	Sensory somatomotor Mouth	R Insula & R BA 13
236	Default mode	R Precuneus
